# Non-Canonical Senescence Phenotype in Resistance to CDK4/6 Inhibitors in ER-Positive Breast Cancer

**DOI:** 10.3390/biom16010093

**Published:** 2026-01-06

**Authors:** Aynura Mammadova, Yuan Gu, Ling Ruan, Sunil S. Badve, Yesim Gökmen-Polar

**Affiliations:** 1 Department of Pathology and Laboratory Medicine, Emory University School of Medicine, Atlanta, GA 30322, USAsbadve@emory.edu (S.S.B.); 2 Winship Cancer Institute, Emory University, Atlanta, GA 30322, USA

**Keywords:** breast cancer, CDK4/6 inhibitors, resistance to cancer therapies, therapy-induced senescence

## Abstract

Cyclin-dependent kinase 4/6 inhibitors (CDK4/6i) have transformed the treatment landscape for estrogen receptor-positive (ER+) breast cancer, yet resistance remains a major clinical challenge. Although CDK4/6i induce G_1_ arrest and therapy-induced senescence (TIS), the exact nature of this senescent state and its contribution to resistance are not well understood. To explore this, we developed palbociclib- (2PR, 9PR, TPR) and abemaciclib- (2AR, 9AR, TAR) resistant ER+ breast cancer sublines through prolonged drug exposure over six months. Resistant cells demonstrated distinct phenotypic alterations, including cellular senescence, reduced mitochondrial membrane potential, and impaired glycolytic activity. Cytokine profiling and enzyme-linked immunosorbent assay (ELISA) validation revealed a non-canonical senescence-associated secretory phenotype (SASP) characterized by elevated growth/differentiation factor 15 (GDF-15) and serpin E1 (plasminogen activator inhibitor-1, PAI-1) and absence of classical pro-inflammatory interleukins, including IL-1α and IL-6. IL-8 levels were significantly elevated, but no association with epithelial–mesenchymal transition (EMT) was observed. Resistant cells preserved their epithelial morphology, showed no upregulation of EMT markers, and lacked aldehyde dehydrogenase 1-positive (ALDH1+) stem-like populations. Additionally, Regulated upon Activation, Normal T-cell Expressed, and Secreted (RANTES) was strongly upregulated in palbociclib-resistant cells. Together, these findings identify a distinct, non-canonical senescence phenotype associated with CDK4/6i resistance and may provide a foundation for identifying new vulnerabilities in resistant ER+ breast cancers through targeting SASP-related signaling.

## 1. Introduction

Cyclin-dependent kinase 4/6 inhibitors (CDK4/6i), palbociclib, ribociclib, and abemaciclib, have introduced a new era in the treatment of estrogen receptor-positive (ER+), HER2-negative metastatic breast cancer. Landmark clinical trials, such as PALOMA, MONALEESA, and MONARCH, have demonstrated significant improvements in progression-free survival when these agents are combined with endocrine therapy in both first- and second-line metastatic settings [1,2]. The MONARCH-2 trial also demonstrated an overall survival benefit, further reinforcing the role of CDK4/6i as standard of care in advanced disease. These findings have established CDK4/6i as a standard of care in advanced ER+ breast cancer [3].

Mechanistically, CDK4/6i exert their therapeutic effect by blocking CDK4/6 and cyclin D complexes and Rb phosphorylation, leading to a G_1_ cell cycle arrest [4]. CDK4/6 inhibition also induces a phenotype resembling cellular senescence in vitro, given the key role of the retinoblastoma (RB) protein in the regulation of senescence. This therapy-induced senescence (TIS) represents a cellular state of persistent cell cycle exit with associated phenotypic alterations [5]. In line with Rb being the main effector of CDK4/6-inhibitor-mediated G_1_ arrest, Rb status also strongly influences senescence induction upon CDK4/6 inhibition [2,6]. CDK4/6 inhibitors thus cause tumor cells to enter an Rb-dependent senescence program with similarities to conventional senescence.

Tissue context and tumor genetic background (p53 status) can influence senescence. CDK4/6 inhibitors induce a senescent state that is dependent on p53 and lacks pro-inflammatory components [7]. Furthermore, active mitogenic signaling appears to set the “depth” of senescence [8,9]. A recent study showed that, if growth signaling (mTORC1) is active during CDK4/6 inhibitor-induced cell cycle arrest, senescence is more pronounced; whereas co-suppression of mTOR attenuates senescence induction [10,11]. In fact, dual mTORC1 inhibition (using everolimus, for example) during palbociclib treatment was reported to prevent “full activation” of senescence [12], consistent with the idea that senescence requires some growth stress (geroconversion) [8,10]. CDK4/6 inhibitors themselves appear to downregulate mTOR activity in breast cancer. As the inflammatory part of the senescent program is controlled by mTOR, this likely impacts the balance between pro- and anti-inflammatory SASP.

TIS is considered a double-edged sword in cancer treatment. On one hand, it acts as a tumor-suppressive mechanism by halting the proliferation of damaged or oncogenic cells in response to treatments such as chemotherapy or radiation. On the other hand, TIS can lead to tumor promotion, reflecting the dual role of senescence in cancer biology through the senescence-associated secretory phenotype (SASP) in a context-dependent manner [13]. The term SASP was first described by Campisi’s group highlighting the complex secretory profile of senescent cells [14]. In response to genotoxic or DNA-damaging stress, senescent tumor cells can secrete a wide array of pro-inflammatory cytokines, such as IL-6, IL-8, growth factors, and proteases [15,16]. By contrast, other forms of non-genotoxic stress have been shown to trigger alternative senescence phenotypes. Campisi’s group described a distinct secretory profile, named mitochondrial dysfunction-associated senescence (MiDAS). It occurs when mitochondrial function is lost or impaired, still resulting in growth arrest and some features of senescence [17]. This metabolic state defined by lower NAD^+^/NADH ratios lacks the IL-1-dependent inflammatory arm of the SASP [17,18]. Instead, in MiDAS, low NAD^+^/NADH ratios activate p53 through AMP-activated protein kinase (AMPK), which then suppresses canonical nuclear factor kappa-light-chain-enhancer (NF-κB)-mediated inflammatory SASP signaling [19]. This distinct secretory profile implicates that senescent cells can be on a spectrum with respect to their secretory phenotypes, ranging from highly tumor-promoting to tumor-suppressive phenotypes.

Despite recent studies exploring the role of CDK4/6 inhibitor-induced senescence, it remains unclear whether senescence directly contributes to therapeutic resistance in the context of CDK4/6 inhibition, as the underlying mechanisms are not yet fully understood. In this study, we developed palbociclib- and abemaciclib-resistant ER+ breast cancer cells (LCC2, LCC9, and T47D) through stepwise increasing exposure to the respective CDK4/6 inhibitors to investigate the nature of senescence in resistance to these therapies. Here, we report a non-canonical SASP phenotype that may play an overlooked role in shaping resistance mechanisms. Future studies should explore the therapeutic potential of targeting specific components of this altered SASP to overcome or prevent resistance to CDK4/6 inhibitor-based therapies.

## 2. Materials and Methods

### 2.1. Cell Culture and Generation of CDK4/6-Resistant Breast Cancer Cell Lines

Parental breast cancer cell lines (LCC2, LCC9, MCF7, and T47D) were grown in Minimum Essential Medium (MEM) with 10% fetal bovine serum (FBS) and 1% penicillin–streptomycin at 37 °C in a humidified 5% CO_2_ incubator. Culture medium was refreshed every 3 days with fresh complete MEM. LCC2 (tamoxifen-resistant) and LCC9 (fulvestrant-resistant and tamoxifen cross-resistant) cell lines were kind gifts from Dr. R. Clarke (Georgetown University Medical School, Washington, DC, USA) [20,21]. MCF7 and T47D cell lines were purchased from American Type Culture Collection (ATCC, Manassas, VA, USA). We developed CDK4/6-resistant sublines of these cell lines through a stepwise increase in palbociclib or abemaciclib drug concentration from 50 nM to 2.5 µM. Cells maintained their ability to proliferate in the presence of palbociclib or abemaciclib. After six months of continuous treatment, drug-resistant sublines were established. Palbociclib-resistant cells of LCC2 (2PR), LCC9 (9PR), and T47D (TPR) were continuously cultured in the presence of 2.5 µM palbociclib, and abemaciclib-resistant cells of LCC2 (2AR), LCC9 (9AR), and T47D (TAR) were cultured in 2.5 µM abemaciclib and used for the experiments described below. Parental (drug-sensitive) LCC2, LCC9, and T47D cells were cultured in parallel under the same conditions but without drug. All cell lines were routinely confirmed to be mycoplasma-free and used at low passage numbers.

### 2.2. Cell Proliferation

Cell viability was assessed using the CyQuant Cell Proliferation Assay (#C35011) according to the manufacturer’s instructions (ThermoFisher Scientific, Waltham, MA, USA). Palbociclib and abemaciclib were obtained from Selleck Chemicals LLC (Houston, TX, USA). Briefly, cells were seeded into 96-well flat-bottom culture plates at 2000 cells per well and incubated overnight. Cells were treated with palbociclib or abemaciclib at various concentrations ranging from 100 nM to 2000 nM for 72 h. At the end of the treatment time, we added the CyQUANT reagent, which contains the dye and a membrane permeabilization agent according to the protocol. Fluorescence was measured using a plate reader (BioTek Synergy H1, Agilent, Santa Clara, CA, USA) with excitation at 485 nm and emission at 530 nm. The relative fluorescence units (RFU) were recorded, providing a quantitative measure of cell proliferation. GraphPad Prism 10.3.1 software was used for the IC50 analysis. CyQUANT measures cell proliferation based on nucleic acid content, which correlates with viable cell number, rather than directly measuring metabolic activity, like the MTT assay.

### 2.3. Senescence Analysis

Senescence-associated β-galactosidase (SA-β-gal) activity was measured using the CellEvent Senescence Green Flow Cytometry Assay (ThermoFisher), according to the manufacturer’s protocol. Flow cytometric analysis was performed using BD FACSDiva v1.01 software. The proportion of senescent cells (green fluorescence) in resistant or parental cell populations was quantified to assess senescence induction by CDK4/6 inhibitors.

### 2.4. NAD^+^/NADH Quantification

Intracellular levels of NAD^+^ and NADH were measured using a fluorometric cycling assay (NAD^+^/NADH Assay Kit, Cell Biolabs, Inc. Cat# MET-5030, San Diego, CA, USA) per the manufacturer’s protocol. Briefly, 4 × 10^6^ cells per sample were harvested and washed with cold PBS before extraction in 0.5 mL of extraction buffer (included in the kit) on ice. Lysates were clarified by centrifugation (5 min, 14,000× *g*, 4 °C) and deproteinated with a 10 kDa molecular-weight cutoff spin filter to remove any enzymes that could degrade NAD. Two aliquots of each sample were prepared and treated differently to distinguish between oxidized and reduced nucleotides. One aliquot was incubated in 0.1 N HCl at 80 °C for 60 min to degrade NAD^+^ (reporting NADH levels), while the other was incubated in 0.1 N NaOH at 80 °C for 60 min to degrade NADH (reporting NAD^+^ levels). Following neutralization and cooling, treated extracts were combined with cycling enzyme, NAD^+^ substrate, and the kit’s fluorescent probe in a black 96-well plate. The enzyme cycling reaction converts NAD^+^ to NADH, which then reacts with the fluorometric probe to generate a fluorescent signal proportional to the NAD concentration. Fluorescence intensity was measured on a plate reader using 530 nm excitation and 590 nm emission settings. Absolute concentrations of NAD^+^ and NADH were determined by comparing to a NAD^+^ standard curve generated in parallel, and the NAD^+^/NADH ratio was calculated for each sample. At least triplicate independent samples were assessed for each condition.

### 2.5. Mitochondrial Membrane Potential Assay (TMRE)

Mitochondrial membrane potential was measured using a tetramethylrhodamine ethyl ester (TMRE) fluorescence assay (TMRE-Mitochondrial Membrane Potential Assay Kit, Abcam, Cat# ab113852, Waltham, MA, USA). Cells were plated into black-walled 96-well plates (clear bottom) at appropriate densities and allowed to attach overnight. On the day of the assay, cells were incubated with TMRE working solution (200 nM final concentration in culture medium) for 30 min at 37 °C in the dark. After incubation, the TMRE-containing medium was carefully removed, and cells were washed twice with warm PBS with 0.2% BSA to reduce background fluorescence. Fluorescence was then measured immediately in a microplate reader (bottom-read mode) with 549 nm excitation/575 nm emission filters, which detects the accumulation of TMRE in polarized mitochondria. A subset of cells was pre-treated with the protonophore carbonyl cyanide-p-trifluoromethoxyphenylhydrazone (FCCP) (20 µM, added 10 min before TMRE incubation) in each experiment as a positive control to depolarize the mitochondrial membrane, which is expected to result in loss of the TMRE signal. Unstained control wells (containing no TMRE dye) were included in all experiments to account for background autofluorescence. Triplicate wells were measured for each condition, and the assay was repeated in at least three independent experiments.

### 2.6. Agilent Seahorse XFp Cell Energy Phenotype Assays

Mitochondrial respiration and glycolysis were assessed using the Agilent Seahorse XF Cell Mito Stress test and Agilent Seahorse XF Glycolysis Stress test, respectively, as previously described [22]. Cells were seeded in XFp miniplates and incubated overnight. The test involved sequential treatment with oligomycin (an ATP synthase inhibitor) and FCCP (a mitochondrial uncoupler) to induce metabolic stress and evaluate cellular energy metabolism. These perturbations enabled the assessment of baseline and stressed energy phenotypes as well as the calculation of metabolic potential by measuring changes in the oxygen consumption rate (OCR) and extracellular acidification rate (ECAR). To fully inhibit mitochondrial respiration and confirm non-mitochondrial oxygen consumption, rotenone (a complex I inhibitor) and antimycin A (a complex III inhibitor) were subsequently injected. This final step ensures accurate quantification of mitochondrial-dependent respiration by subtracting non-mitochondrial OCR from total OCR values. The results presented are the combination of three independent assays, and two-way ANOVA analyses were performed using GraphPad Prism 10.3.1 software (*p* < 0.05, statistically significant).

### 2.7. Cytokine Array

The cytokine array was performed using the Proteome Profiler Array Human XL Cytokine Array Kit (Bio-Techne/R&D Systems, #ARY022B, Minneapolis, MN, USA), according to the manufacturer’s instructions. Imaging and quantification were performed using Amersham Imager 600 software (GE Healthcare Life Sciences, Piscataway, NJ, USA). The signal intensities of six reference spots in each membrane were measured and defined as 100%. A detailed key explaining the protein array layout is provided in the manual for the Proteome Profiler™ Human XL Cytokine Array Kit (Bio-Techne/R&D Systems, catalog #ARY022B).

### 2.8. Cytokine Quantification by ELISA

Protein levels of Interleukin-6 (IL-6, Cat# EH2IL6), Interleukin-8 (IL-8, Cat# KHC0081), Interleukin 1 alpha (IL-1α, Cat# BMS243-2), Regulated Upon Activation, Normal T-cell Ex-pressed, and Secreted (RANTES, Cat# EHRNTS), Angiogenin (Cat# EHANG), Vascular endothelial growth factor (VEGF-A, Cat# KHG0111), and Platelet-derived growth factor-AA (PDGF-AA, Cat# EHPDGFA) in cell culture supernatants were quantified by sandwich ELISA using pre-coated 96-well plate kits (Invitrogen, Thermo Fisher Scientific, Waltham, MA, USA). For each assay, cell culture supernatants were collected after 72 h of incubation (at ~80% cell confluence) and clarified by centrifugation to remove cells and debris. Samples and provided standards were added to the antibody-coated wells and incubated according to the manufacturer’s instructions, followed by addition of biotinylated detection antibodies and horseradish peroxidase (HRP)-conjugated streptavidin. After washing, TMB substrate was added and allowed to develop for the recommended time before the reaction was stopped. Absorbance at 450 nm (reference 540–570 nm) was measured on a microplate reader. Cytokine concentrations in each sample were determined by comparison to the standard curve generated on the same plate. All samples were analyzed in duplicate wells, and each experiment was performed at least in duplicate. Growth/differentiation factor 15 (GDF-15, Cat# DGD150, R&D Systems, Biotechne, Minneapolis, MN, USA), serpin E1 (Plasminogen Activator Inhibitor-1, PAI-1, Cat# DSE100, R&D Systems, Biotechne), and High Mobility Group Box 1 (HMGB1, Cat# 6010, Chondrex, Inc., Woodinville, WA, USA) were similarly measured according to the manufacturer’s instructions.

### 2.9. Morphology Assessment

Cells were grown in 8-well slide chambers (MatTek, Ashland, MA, USA) in CSS media for 4 days. The slides were then fixed in 60% ethanol and stained with hematoxylin and eosin (H&E, Waltham, MA, USA) stains. The slides were examined under an Olympus BX41 microscope (Center Valley, PA, USA), and images were obtained using a DP-72 camera and Olympus cellSens^TM^ Standard 4.3 software at 40× magnification. Morphological assessments were made using 8-well culture plates, with an evaluation of at least 10 (20×) fields by a board-certified breast pathologist (S.B). The analysis was blinded to drug therapy information. The observations were consistent across these fields and in multiple replicates. 

### 2.10. Western Blot Analysis

Protein lysates from control and CDK4/6-resistant cell lines were prepared and quantified using the Bio-Rad DC Protein Assay kit (Bio-Rad, Hercules, CA, USA). Equal protein amounts were analyzed by Sodium Dodecyl Sulfate-Polyacrylamide Gel Electrophoresis (SDS–PAGE) and Western blot as previously described [22]. Briefly, membranes were probed with primary antibodies against Lamin B (Cat# ab16048, Abcam, 0.1 μg/mL); p21 (Cat# 2947, Cell Signaling, dilution 1:1000, Danvers, MA, USA); AMPKα (Cat# 2532, Cell Signaling, dilution 1:100); p-AMPKα (Cat# 2535, Cell Signaling, dilution 1:1000); p16 (Cat# 18769, Cell Signaling, dilution 1:1000); p53 (Cat# 9282, Cell Signaling, dilution 1:1000); NF-κB (Cat# 8242, Cell Signaling, dilution 1:1000); Snail (SNAIL, Cat# 3879 Cell Signaling, dilution 1:1000); Slug (SNAI2, Cat# 9585 (Cell Signaling), dilution 1:1000); TWIST1 (Cat# LS-C342433-100, LS Bio, dilution 1:1000, Newark, CA, USA); and Vimentin (Cell Signaling Technology, Danvers, MA, USA, Cat# 5741 (Cell Signaling), dilution 1:1000). β-actin (Sigma, St. Louis, MO, USA, dilution 1:5000) served as a loading control. Detection was performed using the SuperSignal™ West Pico PLUS Chemiluminescent Substrate kit (Amersham, Piscataway, NJ, USA), and images were captured with the Amersham Imager 600 system (GE Healthcare Life Sciences, Piscataway, NJ, USA). Results shown are representative of three independent experiments. Original figures can be found in Supplementary Materials.

## 3. Results

### 3.1. Prolonged Exposure to Palbociclib or Abemaciclib Confers Resistance in ER+ Breast Cancer Cells

**Figure 1:**
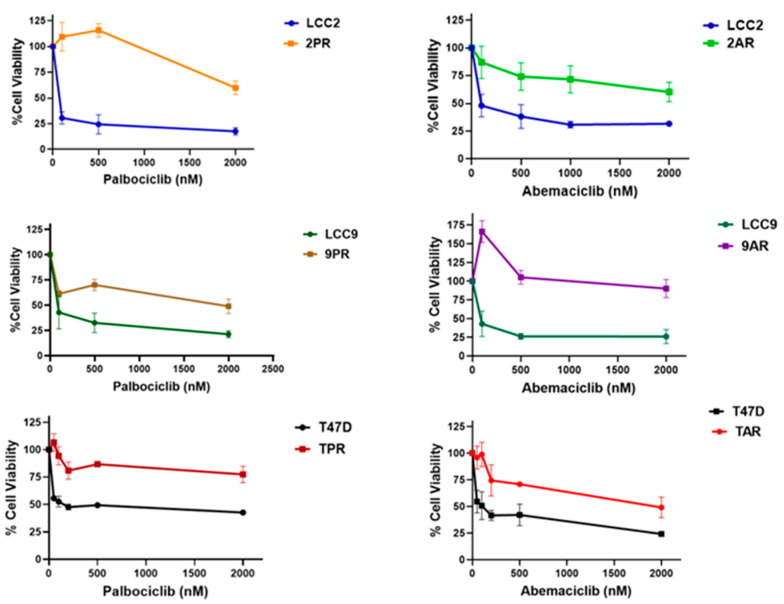
Resistance profile of cells selected in prolonged exposure to palbociclib and abemaciclib. Parental (LCC2, LCC9, and T47D), palbociclib-resistant (2PR, 9PR, and TPR), and abemaciclib-resistant (2AR, 9AR, and TAR) cells were treated for 72 h with increasing concentrations of palbociclib or abemaciclib (CyQuant Cell Proliferation Assay).

The sensitivity to palbociclib and abemaciclib in both parental (LCC2, LCC9, and T47D) and resistant sublines (2PR, 9PR, TPR, 2AR, 9AR, and TAR) was assessed by measuring cell survival rates. Figure 1 shows the comparison of IC_50_ values, representing the concentrations required to reduce cell viability by 50%, for both parental control and resistant sublines. The 2PR, 9PR, and TPR sublines exhibited 645-fold, 57-fold, and 28-fold increases to palbociclib in IC_50_ values compared to their parental sensitive counterparts, respectively. For abemaciclib, the 2AR, 9AR, and TAR sublines displayed 76-fold, 88-fold, and 18-fold increases compared to their parental sensitive counterparts, respectively. This differential response demonstrates that the resistant sublines successfully acquired resistance to each drug.

### 3.2. Palbociclib- and Abemaciclib-Resistant Cells Induce Senescence and Exhibit Markers of Therapy-Induced Senescence

**Figure 2:**
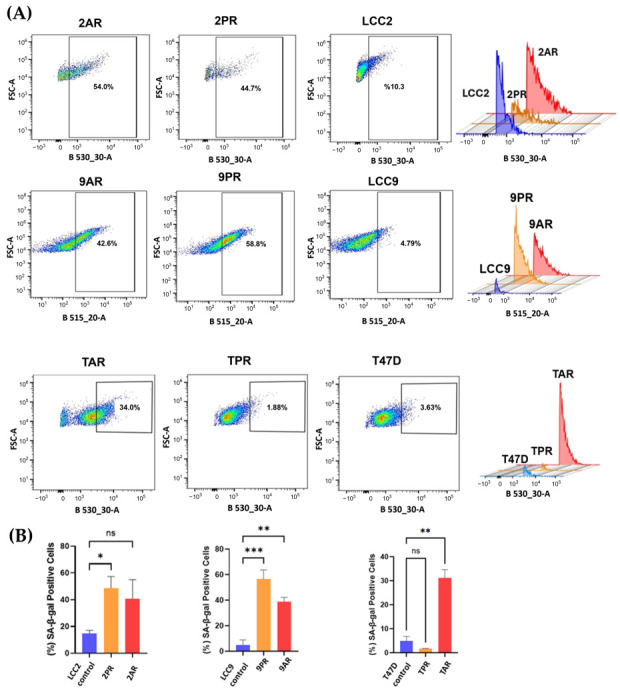
Palbociclib (PR)- and abemaciclib (AR)-resistant cells exhibit increased senescence. Senescence-associated β-galactosidase (SA-β-Gal) activity was measured using flow cytometry with CellEvent™ Senescence Green Kit (Cat# C10840). (**A**) Samples were subjected to CellEvent™ Senescence Green Flow Cytometry Assay (excitation by a 488 nm laser and detection with a 530/30 nm band-pass filter (B530_30-A parameter)). For each sample, 10,000 events were acquired. SA-β-Gal-positive (senescent) cells were quantified by a rectangular gate on FSC-A versus B530_30-A dot plots. The gate was defined by the fluorescence distribution of the parental control sample and applied to the corresponding CDK4/6 inhibitor-resistant sublines. (**B**) Bar plot quantification of the percent of SA-β-Gal-positive cells per group is shown (mean of *n* = 3 of biological replicates). One-way ANOVA test was used to determine significance; * *p* < 0.05; ** *p* < 0.01; *** *p *< 0.001; ns: not significant.

Given the link between CDK4/6 inhibition and therapy-induced senescence (TIS), we evaluated whether the resistant cells adopted a senescent phenotype. To assess this, we measured senescence-associated β-galactosidase (SA-β-Gal) activity, a widely used surrogate marker of cellular senescence. The fluorescence intensity of resistant sublines (2PR, 2AR, 9PR, 9AR, and TAR) exhibited a significant rightward shift compared to their sensitive counterparts, resulting in an increased senescence phenotype, whereas the T47D palbociclib subline (TPR) did not exhibit senescence induction, suggesting differences among the CDK4/6 inhibitors (Figure 2).

Quantification of mean intensities (*n* = 3 of biological replicates) reveals that the senescent fraction of 2PR cells is 48.7%, while 2AR cells display 40.8% positivity for SA-β-Gal, in contrast to the parental LCC2 cells at 10.6% (*p* < 0.0001) (Figure 2B). Specifically, 9PR and 9AR cells exhibited 56% and 39% SA-β-Gal positivity, respectively, versus 5% in parental LCC9 cells, while TPR and TAR cells displayed 2.5% and 31.5% positivity compared with 4.9% in parental T47D cells (*p* < 0.01).

To further explore senescence in CDK4/6i-resistant cells, we first examined the protein expression of established markers, lamin B1 and p21, linked with therapy-induced senescence (TIS) in cancer. Lamin B1, a structural nuclear envelope protein typically downregulated in senescent cells and considered a negative marker of TIS, was significantly reduced in PR sublines (2PR; *p* = 0.0097, and 9PR; *p* = 0.0024) and the 9AR subline (*p* = 0.0462), indicating significant senescence-associated changes (Supplementary Figure S1). We do not see significant altered protein levels in TPR, TAR and 2AR sublines. p21 (Cip1/Waf1), a cyclin-dependent kinase inhibitor regulated by p53 and a key mediator of senescence-associated growth arrest, did not show a significant decrease in 2PR, 2AR, 9PR, 9AR, and TPR, while it was only significantly upregulated in TAR (*p* = 0.0043) sublines, highlighting the heterogeneity of the senescence marker expression across different sublines.

### 3.3. Loss of Mitochondrial Membrane Potential in Resistant Cells

**Figure 3:**
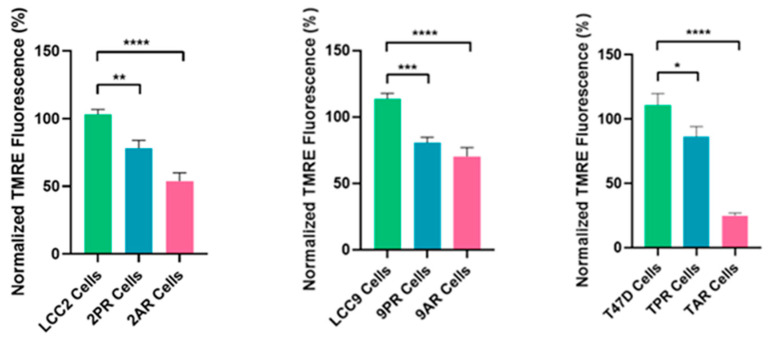
Altered mitochondrial membrane potential in resistant sublines. Mitochondrial membrane potential was assessed by TMRE fluorescence in the same cell lines. Data are shown as a normalized percentage relative to their sensitive parental counterparts. Data represents mean ± SD of at least *n* = 3 independent experiments. One-way ANOVA was used to assess statistical significance, followed by post hoc tests (GraphPad Prism 10.3.1). * *p *< 0.05; ** *p* < 0.01; *** *p *< 0.001; **** *p* < 0.0001. Negative controls are shown in Supplementary Figure S4.

To better define the nature of senescence in palbociclib- and abemaciclib-resistant cells, we next examined mitochondrial dysfunction-associated senescence (MiDAS), as described first by Campisi’s group [17]. As an indication of the MiDAS state, we first measured the NAD^+^/NADH ratio in parental LCC2 cells and their palbociclib- and abemaciclib-resistant derivatives, 2PR and 2AR. Following normalization to LCC2, the mean (±SD) NAD^+^ and the NADH values for 2PR and 2AR were not significantly altered (Supplementary Figure S2). Further analysis of the NAD^+^/NADH ratio also revealed no significant alterations between the resistant sublines and their sensitive counterpart, as determined by one-way ANOVA. Consistent outcomes were observed in parallel assays using LCC9 and T47D parental cell lines and their resistant derivatives (9PR, TPR, 9AR, and TAR), further supporting these observations (Supplementary Figure S2). These results suggest that there is no detectable change in the redox balance in the resistant cells, indicating a lack of substantial mitochondrial dysfunction or metabolic stress. To further evaluate mitochondrial health, we measured mitochondrial membrane potential (ΔΨm) using a TMRE-based assay. This analysis revealed a marked and statistically significant reduction in TMRE intensity in both resistant cell lines compared to LCC2 (Figure 3), indicating a loss of mitochondrial membrane potential (ΔΨm). We verified these results using LCC9 and T47D parental cell lines and their resistant derivatives (9PR, TPR, 9AR, and TAR), further supporting these observations (Supplementary Figure S3). These data suggest that the absence of canonical MiDAS but altered mitochondrial membrane potential in CDK4/6-resistant cells do manifest a different metabolic phenotype, marked by mitochondrial activity impairment.

To further assess the metabolic profile of palbociclib (2PR)- and abemaciclib (2AR)-resistant cells, we performed Seahorse assays to measure key metabolic parameters, including the extracellular acidification rate (ECAR) and the oxygen consumption rate (OCR) (Supplementary Figure S4). Both 2PR and 2AR cells exhibited a marked decrease in ECAR compared to the parental LCC2 cells, suggesting a metabolic shift toward reduced glycolytic capacity and reserve. In addition, 2PR and 2AR cells exhibit significantly reduced mitochondrial function compared to LCC2 cells, as evidenced by lower basal respiration, spare respiratory capacity, and ATP production. Proton leak remains unchanged across comparisons, suggesting that mitochondrial membrane integrity is not notably affected. Similarly, LCC9- and T47D-derived resistant sublines (9PR, 9AR, TPR, TAR) exhibited reduced glycolytic capacity and reserve relative to parental counterparts. Although LCC9-derived resistant cells demonstrated moderate reductions in spare respiratory capacity, they maintained their total mitochondrial function. In contrast, T47D-resistant sublines did not significantly alter any OCR parameters. These observations point to a potential difference in cellular metabolism in the resistant lines and alterations in energy production pathways. The decrease in ECAR further supports the idea that the resistant cells may rely more on oxidative phosphorylation or other alternative metabolic pathways as opposed to glycolysis to meet their energy demands.

### 3.4. SASP Changes in Abemaciclib- and Palbociclib-Resistant Breast Cancer Cells

**Figure 4:**
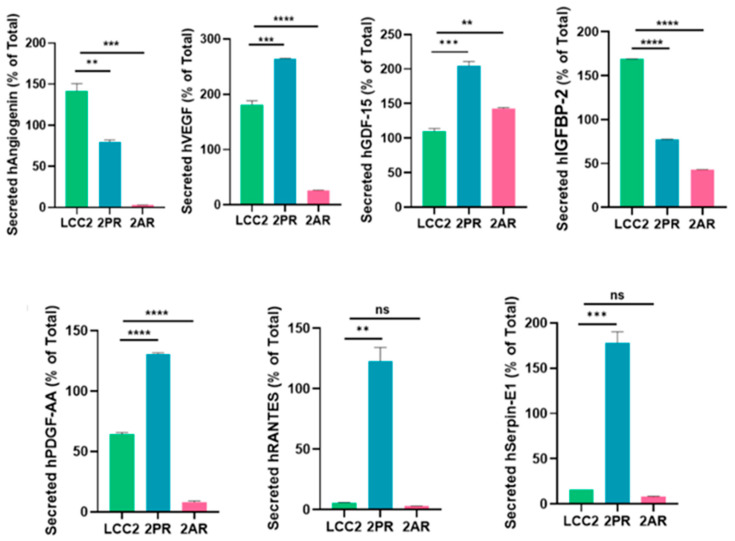
Quantification of significant cytokines/markers using Proteome Profiler Array Human XL Cytokine Array. Conditioned media collected from parental LCC2 cells and CDK4/6 inhibitor-resistant sublines (2PR, resistant to palbociclib, and 2AR, resistant to abemaciclib) were analyzed using a human cytokine antibody array to profile secreted SASP factors. ** *p* < 0.01; *** *p *< 0.001; **** *p *< 0.0001; ns: not significant.

Senescent cells secrete molecules known as the senescence-associated secretory phenotype (SASP), which includes pro-inflammatory cytokines, proteases, and growth and angiogenesis factors [23]. These factors can contribute to therapy resistance. To better understand the role of senescence in 2PR- and 2AR-resistant cells compared to their parental sensitive counterpart (LCC2), we next performed a human cytokine array consisting of key SASP components. Among the factors analyzed, we observed a significant increase in growth/differentiation factor 15 (GDF-15) levels in conditioned media of 2AR and 2PR cells, while angiogenin was dramatically decreased in both resistant cells compared to parental cells (Figure 4; Supplementary Figure S5). Additionally, elevated secretion of VEGF, PDGF-AA, RANTES, and serpin E1 was observed in 2PR cells. Insulin Like Growth Factor Binding Protein 2 (IGFBP-2) levels were significantly decreased in 2PR and 2AR, but signals for IL-6 and IL-1α remained unchanged between resistant and parental cells (Supplementary Figure S5). The complete quantified dataset for all detected proteins is provided as a new supplementary Excel file (Supplementary Tables S1 and S2), which reports normalized expression values for each protein across all samples. These results suggest that resistant cells adopt an altered SASP profile with unique inflammatory and pro-tumorigenic secretions.

### 3.5. ELISA Assays Confirmed Alterations in Cytokine Levels Within the SASP Profiles of Palbociclib- and Abemaciclib-Resistant Breast Cancer Cells

**Figure 5:**
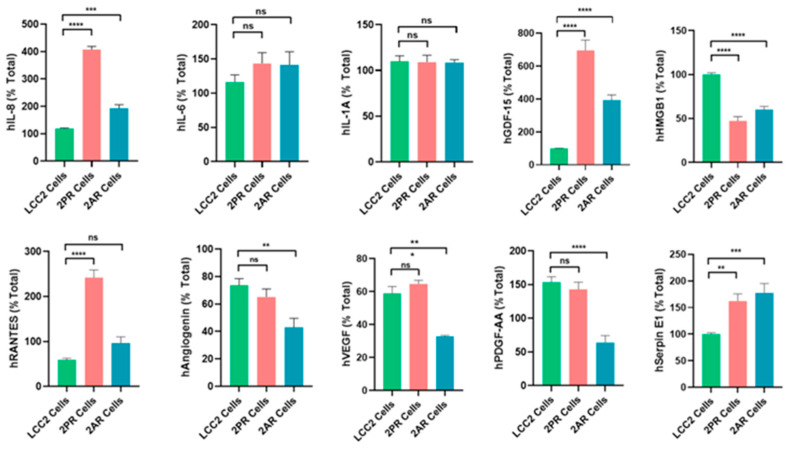
Quantification of altered SASP factors in palbociclib- and abemaciclib-resistant LCC2 sublines compared to their parental cells using ELISA. Supernatants from parental LCC2 (green), LCC2-2PR palbociclib-resistant (red), and LCC2-2AR abemaciclib-resistant (blue) cells were collected and subjected to enzyme-linked immunosorbent assay (ELISA) for a panel of key SASP-associated factors. Ten secreted proteins were quantified: IL-8, IL-6, IL-1α, GDF-15, HMGB1 (high mobility group box 1), RANTES (CCL5), angiogenin, VEGF, PDGF-AA, and serpin E1 (PAI-1). Bar graphs present the mean levels of each factor in the media of the three cell lines (expressed as a percentage of the total level measured for that factor, see Section 2), with error bars indicating mean ± SD of *n* = 3 independent experiments (triplicates each). Significance was determined by one-way ANOVA and post hoc tests: * *p* < 0.05; ** *p* < 0.01; *** *p* < 0.001; **** *p *< 0.0001; ns: not significant.

To validate the array results, we quantitatively measured the levels of key SASP factors in the conditioned medium of 2PR and 2AR sublines compared to parental LCC2 cells by ELISA (Figure 5). These analyses confirmed that IL-8 (2PR, *p* < 0.0001; 2AR, *p* < 0.001), GDF-15 (2PR, *p* < 0.0001; 2AR, *p* < 0.0001), and serpin E1 (PAI-1) (2PR, *p* < 0.01; 2AR, *p* < 0.001) were significantly upregulated in both 2PR and 2AR conditioned media compared to LCC2. RANTES was upregulated only in 2PR cells (*p* < 0.0001). In contrast, secretion of IL-6 or IL-1α was not significantly changed in the resistant cells. Similarly, angiogenin, VEGF, and PDGF-AA levels were not significantly altered in 2PR. In contrast, they were significantly decreased in 2AR cells (angiogenin, *p* < 0.01; VEGF, *p* < 0.01; and PDGF-AA, *p* < 0.0001). We also observed similar alterations of these cytokines/markers in palbociclib- and abemaciclib-resistant derivatives of LCC9 and T47D cells (Supplementary Figure S6).

We also measured other SASP markers that have been reported by the Campisi group to contribute to the senescence-associated arrest, including High-Mobility Group Box 1 (HMGB1), AMP-activated protein kinase (AMPK), nuclear factor kappa-light-chain-enhancer of activated B (NF-κB), and p53 [17]. HGMB1 secretion was significantly reduced in the 2PR (*p* < 0.0001) and 2AR (*p* < 0.0001) conditioned media, supporting that this pro-inflammatory nuclear protein is not involved. In addition, AMPK, phospho-AMPK, p16, and p53 are not altered/not detected in the resistant cells, as shown in Supplementary Figure S7. Overall, these results demonstrate that LCC2 cells selected for CDK4/6 inhibitor resistance acquire a distinct SASP-like secretory phenotype, which is characterized by the marked upregulation of some pro-inflammatory cytokines (IL-8) but not the classical inflammatory cytokines, including IL1-α and IL-6. Consistent with these results, NF-κB levels, the major pathway activated by IL1-α, were significantly downregulated in 2PR and not altered in any other resistant sublines compared to their sensitive counterparts. Moreover, p53, another factor commonly associated with senescence, was not involved in these resistant cell lines, suggesting that the mechanism underlying resistance is p53-independent. We also observed similar alterations of these cytokines/markers in palbociclib- and abemaciclib-resistant derivatives of LCC9 and T47D cells. Taken together, these studies indicate that CDK4/6 inhibitor-resistant ER-positive breast cancer cells are characterized by a non-canonical inflammatory secretome rather than a classical pro-inflammatory cytokine profile.

### 3.6. Resistance to CDK4/6 Inhibitors Does Not Promote EMT Phenotype in ER+ Breast Cancer Models

**Figure 6:**
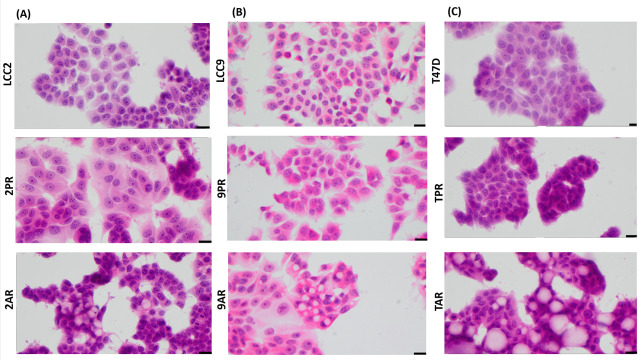
Palbociclib- and abemaciclib-resistant cells do not exhibit morphological features characteristic of EMT. Cell morphology using high-power magnification (40x) of the cultured cell lines under a microscope in (**A**) LCC2 model; 2PR (palbociclib-resistant subline of LCC2), 2AR (abemaciclib-resistant subline of LCC2), and LCC2 (CDK4/6i-sensitive parental cell line). Scale bar: 20 µm. (**B**) LCC9 model; 2PR (palbociclib-resistant subline of LCC2, abemaciclib-resistant subline of LCC2) and LCC9 (CDK4/6i-sensitive parental cell line). Scale bar: 20 µm. (**C**) T47D model; TPR (palbociclib-resistant subline of LCC2), TAR (abemaciclib-resistant subline of LCC2), and T47D (CDK4/6i-sensitive parental cell line). Scale bar: 20 µm.

IL-8 has been implicated in promoting epithelial-to-mesenchymal transition (EMT) in various tumor types, and resistance to therapies is often associated with mesenchymal-like characteristics. To assess whether EMT contributes to resistance in CDK4/6 inhibitor (CDK4/6i) models, we examined the morphology of palbociclib- and abemaciclib-resistant breast cancer cells. All resistant models (2PR, 9PR, TPR, 2AR, 9AR, and TAR) maintained an epithelial morphology, with no evidence of mesenchymal-like changes (Figure 6).

To further evaluate EMT status, we analyzed the expression of canonical EMT transcription factors using Western blotting (Supplementary Figure S8). Expression levels of the EMT inducers Slug (SNAI2), Snail (SNAI1), and TWIST1 were not significantly altered in either resistance model. Vimentin, a key mesenchymal cytoskeletal marker, was undetectable in both palbociclib- and abemaciclib-resistant cells. These findings indicate that resistance to CDK4/6 inhibitors does not involve EMT, and resistant cells do not acquire a mesenchymal phenotype. To assess whether stem-like properties contribute to resistance, we also evaluated ALDH1 activity—a recognized marker of breast cancer stem cells—using the ALDEFLUOR assay. No ALDH1 activity was detected in CDK4/6 inhibitor-resistant cells (Supplementary Figure S9), providing no evidence to support a role for ALDH1-positive stem-like cells in resistance.

## 4. Discussion

We show that CDK4/6 inhibitor-resistant ER+ breast cancer cells display a non-canonical, metabolically altered senescence phenotype marked by loss of mitochondrial membrane potential and a selectively altered SASP profile. This distinct phenotype underscores the atypical nature of these resistant cells and calls into question conventional definitions of senescence. Conventionally, therapy-induced senescence (TIS) in cancer cells has been characterized by permanent growth arrest coupled with a pro-inflammatory senescence-associated secretory phenotype (SASP) enriched in key cytokines like IL-6 and IL-8 as well as various growth factors [24]. IL-1α is generally thought to be required for the amplification of the SASP via the NF-κB pathway [25], and its absence here could indicate a selective suppression or rewiring of upstream regulators in the resistant cells.

Besides the IL-1/NF-κB-dependent secretome, Campisi’s group further categorized senescent phenotypes into mitochondrial dysfunction-associated senescence (MiDAS) secretome using a fibroblast model system. They also demonstrated MiDAS as another form of cellular senescence that may play a role in age-related diseases, where it was observed in mouse models of progeria, and affect metabolism [17]. Narita’s group focused on the temporal and spatial aspects of senescence and emphasized that the role of senescence is time dependent [26,27,28,29]. They further posit that SASP in early stages promotes tissue repair, while in later stages, it helps recruit immune cells for senescence clearance. They postulate an important role of the NOTCH pathway in the process.

In our study, the CDK4/6 inhibitor-resistant breast cancer models diverged significantly from these senescence programs described above. Specifically, we do not observe significant and consistent alterations in p21 and p16, which are downstream effectors of NOTCH. Similarly, the lack of alteration of key SASP components, such as IL-6, IL-1α, and NF-κB, in these resistant cells points to a senescent phenotype that is distinct from this pro-inflammatory senescence arrest. Significant downregulation of secreted HMGB1, a key mediator of senescence-associated inflammation [30], does not favor inflammatory signaling in our models. Additionally, we did not observe the expected decrease in the NAD^+^/NADH ratio typically associated with MiDAS. Instead, our study revealed an altered cellular metabolism marked by significant loss of mitochondrial membrane potential and a decrease in ATP production via oxidative phosphorylation (OXPHOS) in resistant sublines, indicating a distinct altered cellular metabolism. These findings together suggest the presence of mitochondrial stress and reflect an adaptive mechanism by shifting energy balance or altering signaling pathways to bypass drug-induced cell cycle arrest. This state could signal a non-canonical senescence phenotype, where cells remain metabolically active but do not conform to traditional senescence markers, including p16 and p53, or AMPK. Although we have not been able find a similar description in cancer cells undergoing senescence, Li et al. [31] documented the association of mitochondrial mutations and senescence in patients being treated for HIV. Our observations of mitochondrial dysfunction resulting in altered OXPHOS without alterations in NAD/NADH ratios need to be further studied to understand its mechanistic basis.

To further understand the SASP in resistant cells, we conducted a detailed analysis of other secretory profiles in resistant cells compared to their sensitive counterparts. The upregulation of SASP factors, including serpin E1 (PAI-1), IL-8, and GDF-15 [25,32,33], but not IL1-α- and IL-6-dependent signaling suggests a non-canonical senescence phenotype characterized by distinct SASP features. An increased level of serpin E1/PAI-1, a multifunctional secreted protein, has been previously implicated in metastasis and stromal remodeling [34]. uPA/PAI-1 is a Level 1 prognostic biomarker in early-stage breast cancer; high levels are associated with poor progression-free and overall survival [35,36]. PAI-1 is linked to enhanced tumor cell survival, invasion, and metastasis, particularly through its interaction with the urokinase plasminogen activator (uPA) system [37]. High PAI-1 mRNA levels predict poor outcomes and shorter overall survival [38,39]. Elevated plasma PAI-1 correlates with a higher relapse risk and worse overall survival [40]. Significant upregulation of PAI-1 was also observed in other therapies, such as Alisertib, an Aurora kinase inhibitor [41]. These findings support that PAI-1 is not only a marker of senescence but also a maintainer of the senescent phenotype under therapeutic stress. Its selective induction in aggressive breast cancer models following senescence activation further suggests a functional role in therapy-induced senescence.

We also observed an increased level of GDF-15, a member of the transforming growth factor-beta (TGF-β) superfamily that has been upregulated in response to cellular stress, including mitochondrial dysfunction, oxidative stress, and inflammation [42]. Other studies reported that GDF-15 overexpression is linked to aggressive phenotypes, radio-resistance, poor response to chemotherapy, and failure of immune checkpoint inhibitors (ICIs) in cancer, including head and neck squamous cell carcinoma and breast cancer [43,44]. GDF-15 is upregulated in breast cancer tissues, especially in drug-tolerant persister (DTP) cells, which are linked to treatment resistance and recurrence [45].

IL-8 (CXCL8), another upregulated chemokine in our CDK4/6 inhibitor-resistant sublines, plays a critical role in tumor angiogenesis, metastasis, and chemoresistance, contributing to cancer progression [46]. Elevated IL-8 levels at baseline are linked to poor overall survival in breast cancer patients undergoing chemotherapy. Notably, the observed upregulation of IL-8 without corresponding upregulation of IL-1α is intriguing. A remodeled SASP may represent a key element of the senescence phenotype that allows for tumor progression.

The non-canonical senescence state that we have described here also opens the possibility that senescence is not an end-stage process in cancer and can persist in tumors. Several recent reports show that senescent tumor cells can acquire stem-like properties and re-enter the cell cycle, contributing to tumor recurrence [47]. The secretory phenotype of these drug-resistant cells and their dependence on the selective secretion of IL-8 and other inflammatory mediators could facilitate such plasticity in adjacent cells in a manner consistent with the senescence-associated stemness phenotype recently described in lymphoma and colorectal cancer models [43]. Despite the upregulation of IL-8, we do not see an epithelial–mesenchymal transition (EMT) phenotype or any upregulation of EMT-TFs as well as ALDH activity, a stem cell marker in breast cancer. This observation is consistent with clinical settings in which a clear role for EMT has been questioned in ER+ breast cancer patient samples [48].

## 5. Conclusions

This study suggests a distinct, non-canonical senescence mechanism employed by CDK4/6 inhibitor-resistant breast cancer cells to maintain therapeutic resistance. This mechanism is associated with altered metabolic functions and an atypical SASP profile. From a clinical perspective, the senescent state in resistant cells that we describe may provide a useful framework for patient stratification and for exploring targeted treatment strategies. These features could represent potential therapeutic vulnerabilities that, if further validated in a real-world setting, may help improve the efficacy of CDK4/6 inhibitors and inform the development of rational combination therapies aimed at targeting or modulating senescent cell populations.

## Data Availability

The original contributions presented in this study are included in the article/Supplementary Material. Further inquiries can be directed to the corresponding author(s).

## Supplementary Materials

The following supporting information can be downloaded at: https://www.mdpi.com/article/10.3390/biom16010093/s1. Supplementary Figure S1. Protein levels of established senescence markers (Lamin B1 and p21) in parental and CDK4/6 inhibitor–resistant cells. Supplementary Figure S2. Cellular NAD+/NADH ratio in resistant sublines. Supplementary Figure S3. Validation of the TMRE assay using FCCP as a negative control in parental and CDK4/6 inhibitor–resistant breast cancer cell lines. Supplementary Figure S4. Seahorse Metabolic Analyses on Palbociclib and Abemaciclib-resistant sublines. Supplementary Figure S5. The SASP protein expression profiling in parental LCC2 cells compared to CDK4/6 inhibitor-resistant LCC2 cells. Supplementary Figure S6. Quantification of altered SASP factors in palbociclib and abemaciclib- resistant LCC9 and T47D sublines compared to their parental counterparts using ELISA. Supplementary Figure S7. Protein levels of markers that may play a role in SASP. Supplementary Figure S8. Expression of EMT- Transcription Factors in parental and CDK4/6 inhibitor–resistant sublines of ER+ breast cancer. Supplementary Figure S9. ALDH+ populations are not changed in resistant cells compared to their parental counterparts. Supplementary Table S1. Quantification of proteins in Proteome Profiler Array Human XL Cytokine Array Kit (Bio-Techne/R&D Systems, #ARY022B), palbocilib-resistant 2PR and LCC2 control. Supplementary Table S2. Quantification of proteins in Proteome Profiler Array Human XL Cytokine Array Kit (Bio-Techne/R&D Systems, #ARY022B), abemaciclib-resistant 2AR and LCC2 control.

## Abbreviations

The following abbreviations are used in this manuscript:

ALDH1	Aldehyde dehydrogenase 1
AMPK	AMP-activated protein kinase (AMPK)
CDK4/6i	CDK4/6 inhibitors
ECAR	Extracellular acidification rate
ELISA	Enzyme-linked immunosorbent assay
EMT	Epithelial–mesenchymal transition
ER+	Estrogen receptor-positive
FBS	Fetal bovine serum
FCCP	Carbonyl cyanide-p-trifluoromethoxyphenylhydrazone
GDF-15	Growth/differentiation factor 15
H&E	Hematoxylin and eosin
HMGB1	High mobility group box 1 protein
IGFBP-2	Insulin like growth factor binding protein 2
IL-1α	Interleukin-1alpha
IL-6	Interleukin-6
IL-8	Interleukin-8
MEM	Minimum Essential Medium
MiDAS	Mitochondrial dysfunction associated senescence
NF-κB	Nuclear factor kappa-light-chain-enhancer of activated B
OCR	Oxygen consumption rate
OXPHOS	Oxidative phosphorylation
PAI-1	Plasminogen activator inhibitor-1
PDGF-AA	Platelet-derived growth factor AA
RANTES	Regulated upon activation, normal T-cell expressed, and secreted
SA-β-Gal	Senescence-associated β-galactosidase
SASP	Senescence-associated secretory phenotype
SDS-PAGE	Sodium dodecyl sulfate-polyacrylamide gel electrophoresis
TIS	Therapy-induced senescence
TMRE	Tetramethylrhodamine ethyl ester
VEGF	Vascular endothelial growth factor
2PR	Palbociclib-resistant LCC2 subline
9PR	Palbociclib-resistant LCC9 subline
TPR	Palbociclib-resistant T47D subline
2AR	Abemaciclib-resistant LCC2 subline
9AR	Abemaciclib-resistant LCC9 subline
TAR	Abemaciclib-resistant T47D subline
