# Supplementary material for: Non-Canonical Senescence Phenotype in Resistance to CDK4/6 Inhibitors in ER-Positive Breast Cancer
Source: Biomolecules. 2026 Jan 6;16(1):93. doi: 10.3390/biom16010093 (PMC12838639; doi:10.3390/biom16010093)
Supplement: Supplementary file 1 [file biomolecules-16-00093-s001.zip › Supplementary Materials_revised version_1-5-2026.pdf]

Supplementary Materials

Non-Canonical Senescence Phenotype in Resistance to CDK4/6 Inhibitors in ER-Positive Breast Cancer

Aynura Mammadova [1], Yuan Gu [1], Ling Ruan [1], Sunil S. Badve [1,2,†] and Yesim Gökmen-Polar [1,2,*,†]

[1] Department of Pathology and Laboratory Medicine, Emory University School of Medicine, Atlanta, GA 30322, USA; sbadve@emory.edu (S.S.B.)
[2] Winship Cancer Institute, Emory University, Atlanta, GA 30322, USA
* Correspondence: ypolar@emory.edu
† These authors contributed equally to this work.

The following supporting information can be downloaded at: https://www.mdpi.com/article/doi/s1.

Supplementary Figure S1. Protein levels of established senescence markers (Lamin B1 and p21) in parental and CDK4/6 inhibitor–resistant cells.

Supplementary Figure S2. Cellular NAD+/NADH ratio in resistant sublines.

Supplementary Figure S3. Validation of the TMRE assay using FCCP as a negative control in parental and CDK4/6 inhibitor–resistant breast cancer cell lines.

Supplementary Figure S4. Seahorse metabolic analyses on palbociclib and abemaciclib-resistant sublines.

Supplementary Figure S5. The SASP protein expression profiling in parental LCC2 cells compared to CDK4/6 inhibitor-resistant LCC2 cells.

Supplementary Figure S6. Quantification of altered SASP factors in palbociclib and abemaciclib-resistant LCC9 and T47D sublines compared to their parental counterparts using ELISA.

Supplementary Figure S7. Protein Levels of Markers Potentially Involved in SASP.

Supplementary Figure S8. Expression of EMT- Transcription Factors in parental and CDK4/6 inhibitor–resistant sublines of ER+ breast cancer.

Supplementary Figure S9. ALDH+ populations are not changed in resistant cells compared to their parental counterparts.

Supplementary Table S1. Quantification of proteins in Proteome Profiler Array Human XL Cytokine Array Kit (Bio-Techne/R&D Systems, #ARY022B), palbocilib-resistant 2PR and LCC2 control.

Supplementary Table S2. Quantification of proteins in Proteome Profiler Array Human XL Cytokine Array Kit (Bio-Techne/R&D Systems, #ARY022B), abemaciclib-resistant 2AR and LCC2 control.

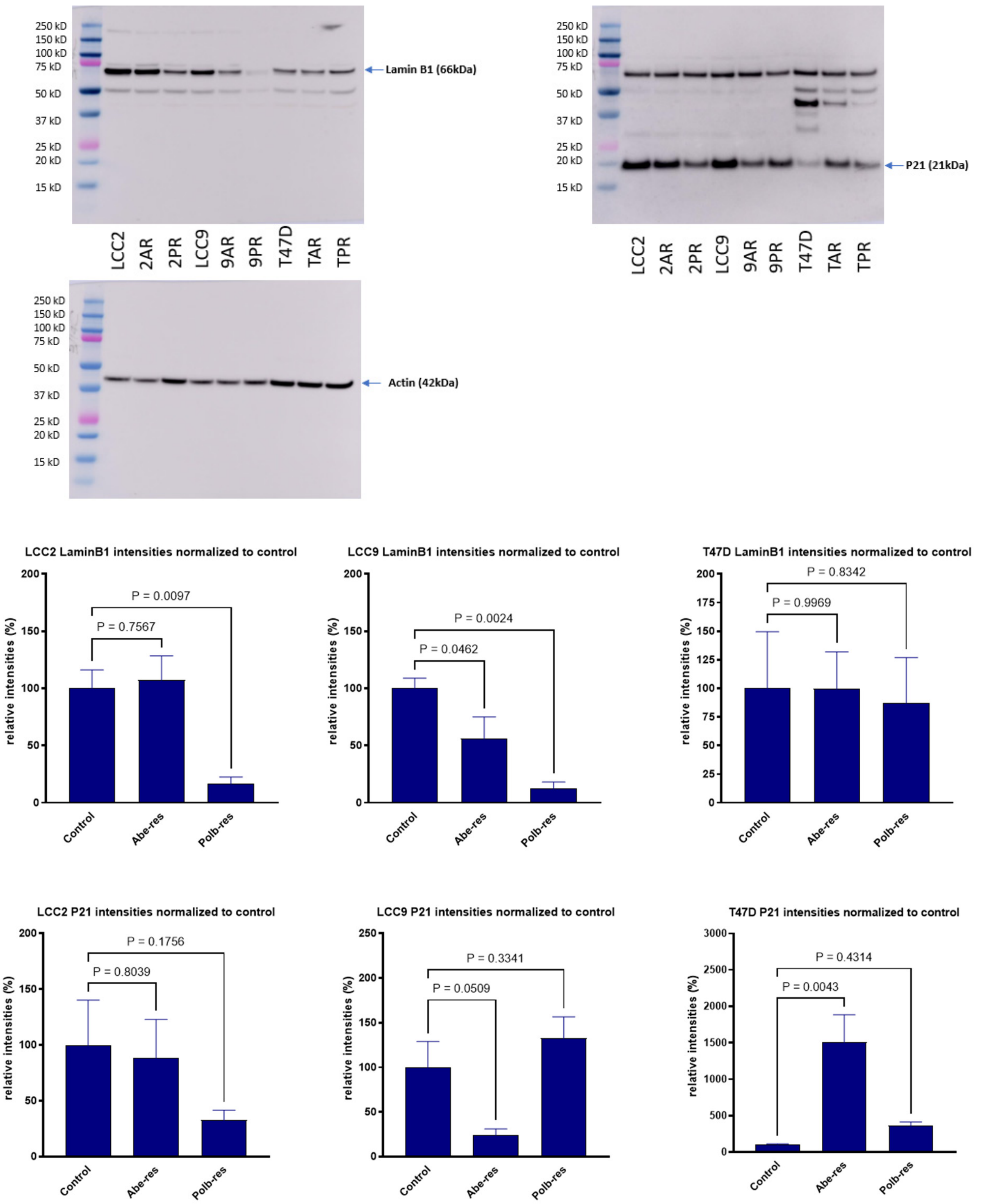

Supplementary Figure S1. **Protein levels of established senescence markers (Lamin B1 and p21) in parental and CDK4/6 inhibitor–resistant cells.** The original unprocessed Western blots for protein expression in abemaciclib-resistant (AR) and palbociclib-resistant (PR) sublines were shown above. Protein molecular weight marker was purchased from BIO-RAD-Precision Plus Protein Dual Color Standards (Catalog #1610374, BIO-RAD, Hercules, CA, USA). Quantification data of Lamin B1 and p21 protein levels in PR and AR cells are presented as mean ± SD from three independent biological replicates and were analyzed using ordinary one-way ANOVA (GraphPad Prism 10.3.1); β-actin (Sigma, St. Louis, MO, USA) was used as the reference control. Cells: Parental (LCC2, LCC9 and T47D), palbociclib-resistant (2PR, 9PR and TPR), and abemaciclib-resistant (2AR, 9AR and TAR).

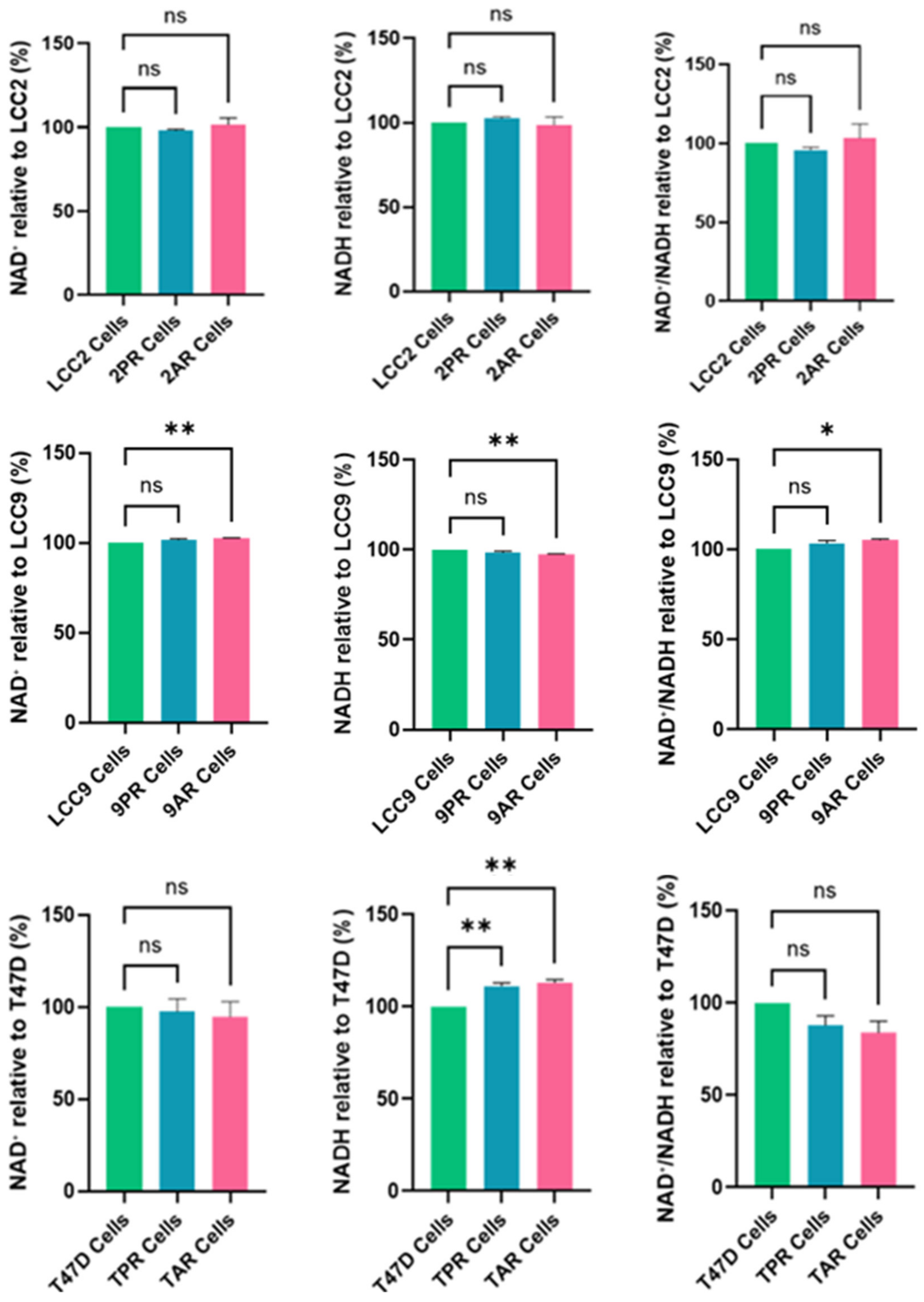

Supplementary Figure S2. Cellular NAD+/NADH ratio in resistant sublines. NAD+ and NADH levels were quantified and expressed as % of total NAD+/NADH in LCC2, LCC9 and T47D parental cells, palbociclib-resistant (2PR, 9PR and TPR), and abemaciclib-resistant (2PR, 9AR and TAR) cells. Data are shown as a normalized percentage relative to their parental cells. Data represents mean ± SD of at least $n = 3$ independent experiments. One-way ANOVA was used to assess statistical significance followed by post hoc tests (GraphPad Prism 10.3.1). * $p < 0.05$; ** $p < 0.01$; ns: not significant.

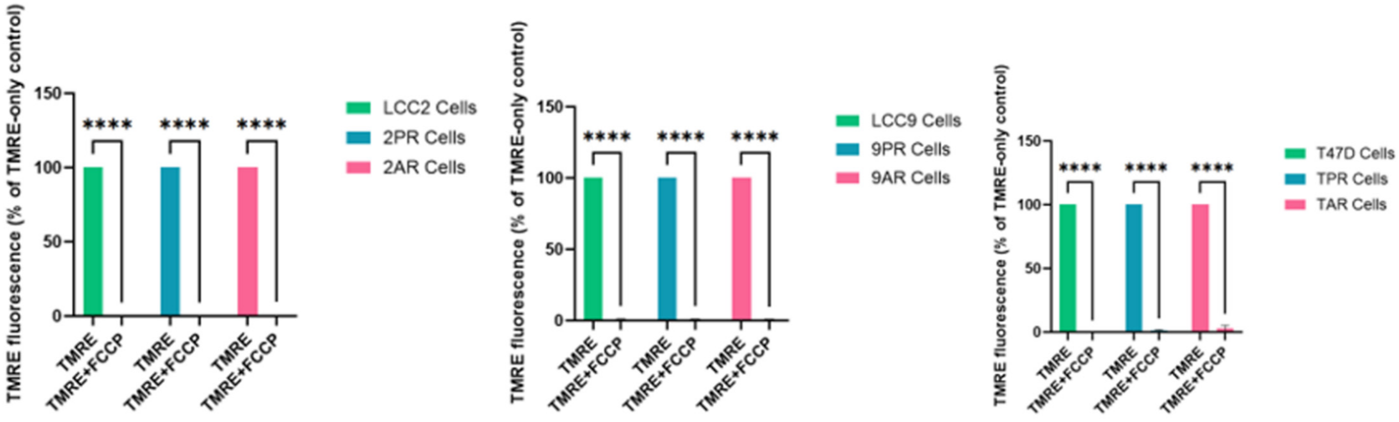

Supplementary Figure S3. Validation of the TMRE assay using FCCP as a negative control in parental and CDK4/6 inhibitor–resistant breast cancer cell lines. TMRE fluorescence in the presence of FCCP is expressed as % of the TMRE-only signal after background subtraction (mean ± SEM, $n = 3$ independent experiments). Values close to 0% indicate almost complete loss of mitochondrial membrane potential (ΔΨm). Cells: LCC2 set (LCC2, 2PR and 2AR cells), LCC9 set (LCC9, 9PR and 9AR cells) and T47D set (T47D, TPR and TAR cells). One-way ANOVA was used to assess statistical significance, followed by post hoc tests (GraphPad Prism 10.3.1). **** $p < 0.0001$.

(A)

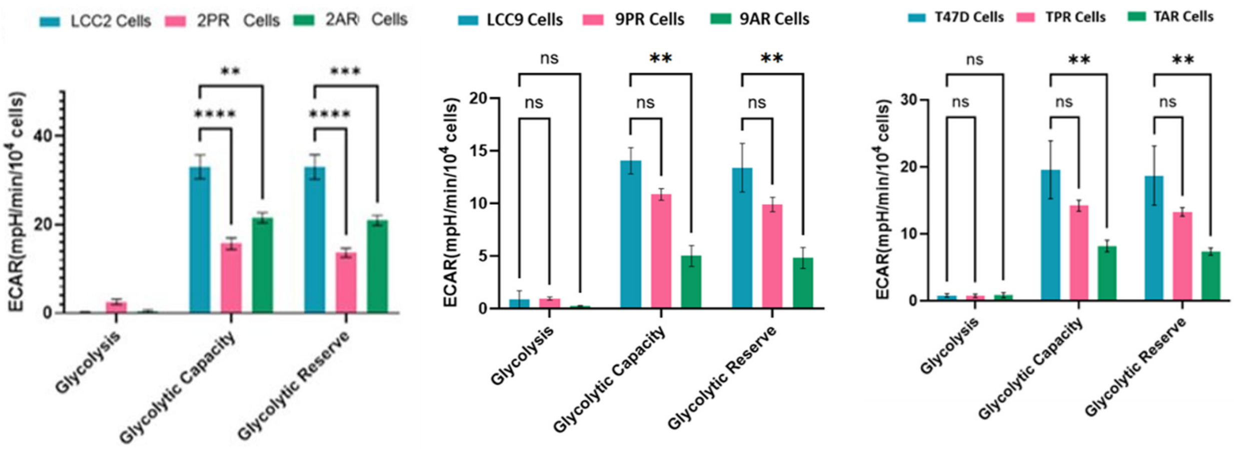

(B)

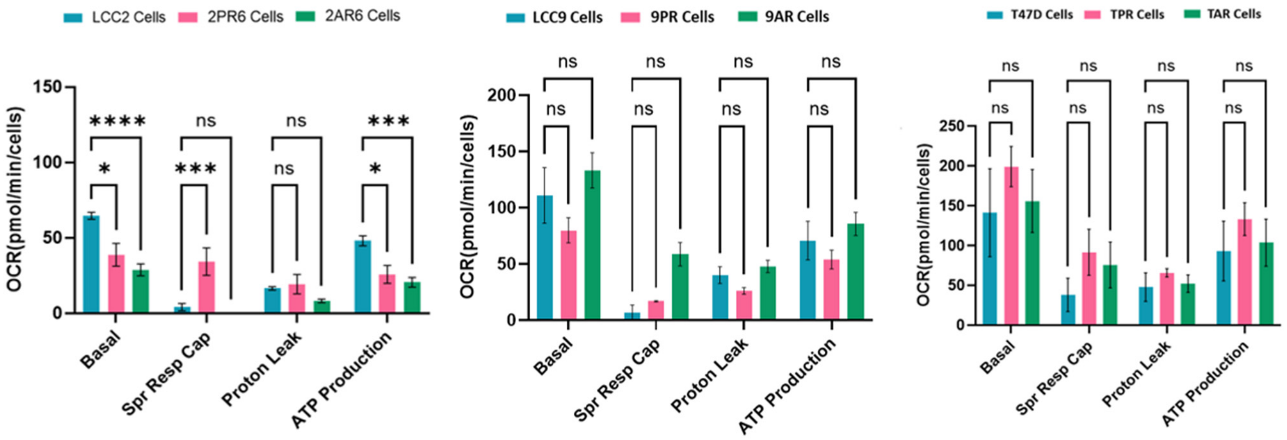

Supplementary Figure S4. Seahorse metabolic analyses on palbociclib and abemaciclib -resistant sublines. (A) Glycolytic function by Seahorse XF Glycolysis Stress Test (ECAR). Seahorse XF analysis was performed on parental (LCC2, LCC9 and T47D), palbociclib-resistant (2PR, 9PR and TPR), and abemaciclib-resistant (2AR, 9AR and TAR) cells; Bars depict Glycolysis, Glycolytic Capacity, and Glycolytic Reserve (mpH/min/10^4 cells; mean ± SD; n = 3–4 wells per experiment, ≥3 independent experiments). One-way ANOVA with Tukey's post hoc test. **(B)** Mitochondrial respiration by Seahorse XF Cell Mito Stress Test (OCR). Bars for Basal respiration, Spare Respiratory Capacity, Proton Leak, and ATP-linked OCR (pmol O_2/min/10^4 cells; mean ± SD; n = 3–4 wells, ≥3 independent experiments). One-way ANOVA with Tukey's post hoc test.

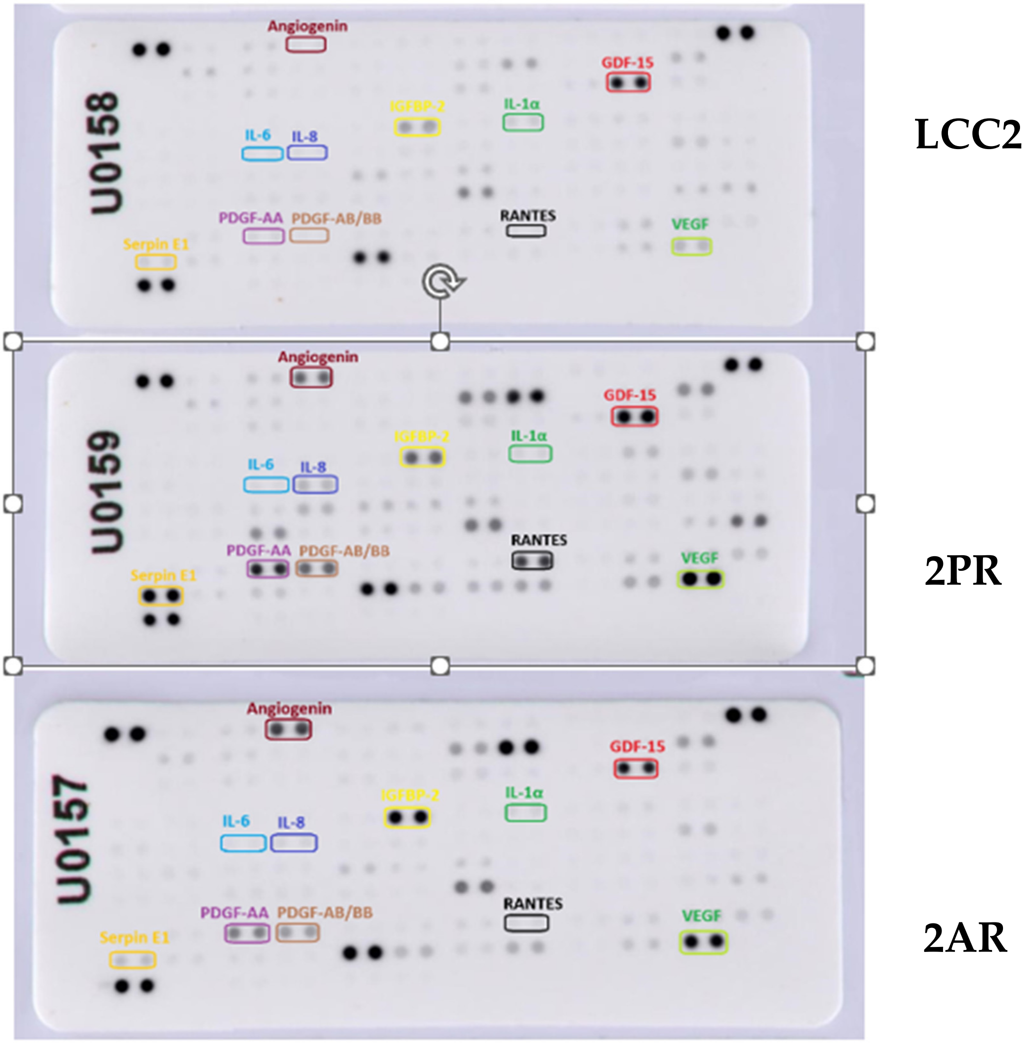

Supplementary Figure S5. The SASP protein expression profiling in parental LCC2 cells compared to CDK4/6 inhibitor-resistant LCC2 cells. Conditioned media from parental LCC2 cells and CDK4/6 inhibitor–resistant sublines (2PR, Palbociclib-resistant; and 2AR, Abemaciclib-resistant) were applied to a human cytokine antibody array to profile secreted SASP factors. Each membrane shows representative array membranes for LCC2, 2PR, and 2AR, respectively. Dots on the arrays (in duplicate) represent individual secreted proteins, with spot intensity corresponding to the relative protein abundance.

(A)

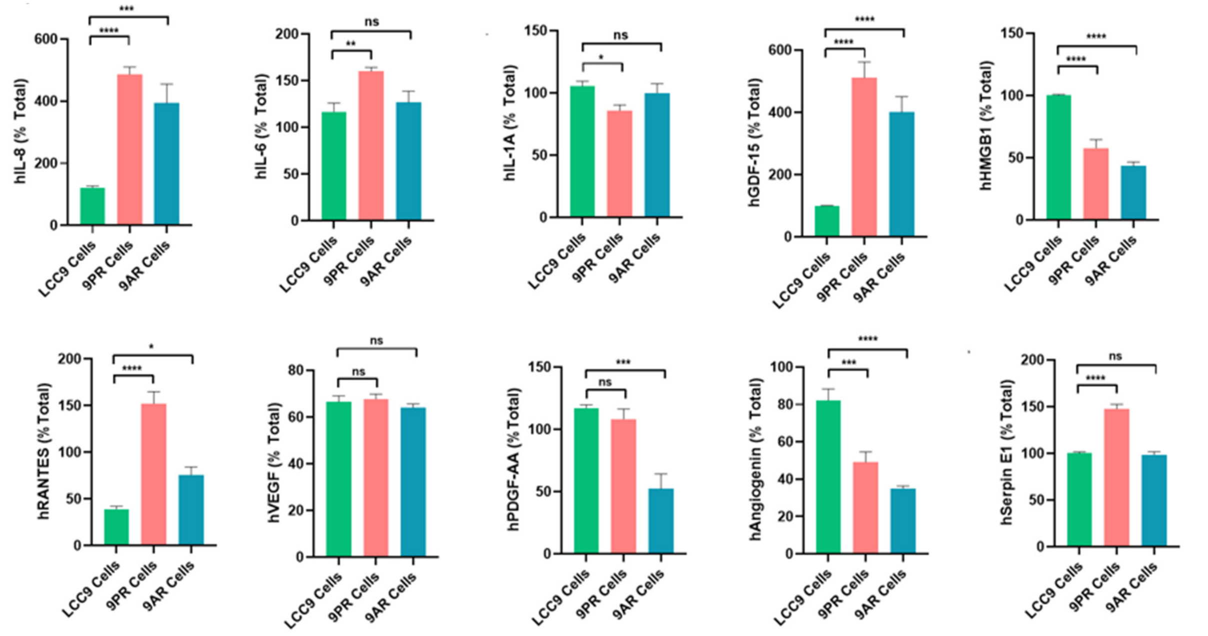

(B)

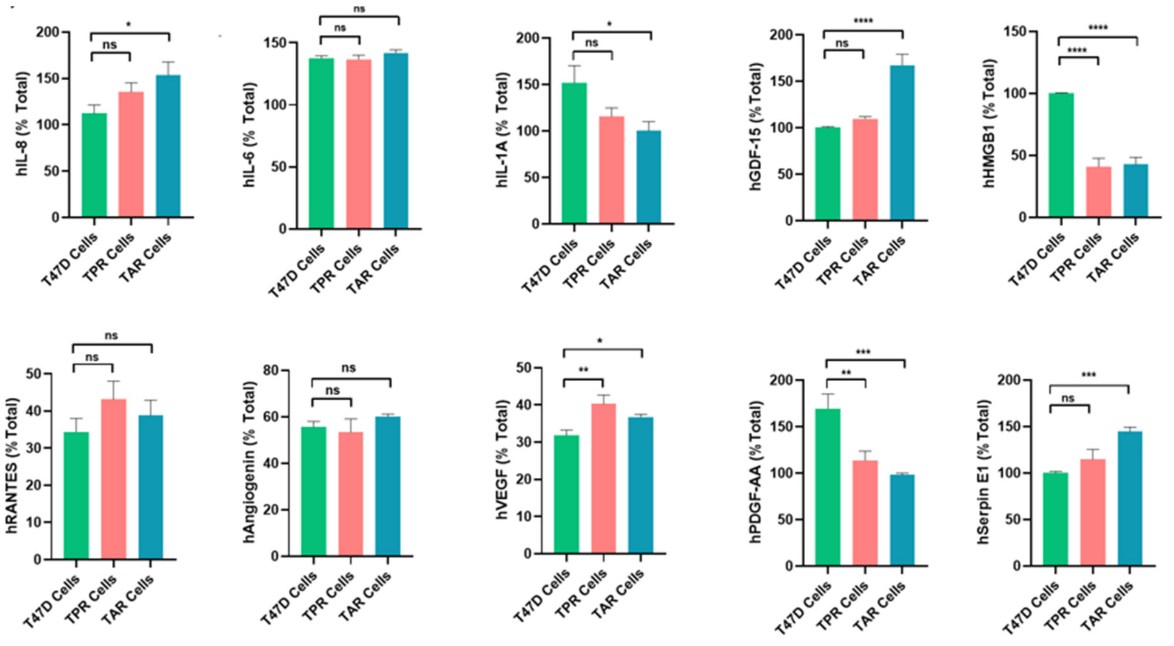

Supplementary Figure S6. Quantification of altered SASP factors in palbociclib and abemaciclib-resistant LCC9 and T47D sublines compared to their parental counterparts using ELISA. Supernatants from **(A)** LCC9, 9PR (palbociclib-resistant), and 9AR (abemaciclib-resistant) cells and **(B)** T47D, TPR (palbociclib-resistant) and TAR (abemaciclib-resistant) cells were collected and subjected to enzyme-linked immunosorbent assay (ELISA) for a panel of key SASP-associated factors. The following secreted cytokines/markers were quantified: IL-8, IL-6, IL-1α, GDF-15, HMGB1 (high mobility group box 1), RANTES (CCL5), angiogenin, VEGF, PDGF-AA, and serpin E1 (PAI-1). Bar graphs present the mean levels of each factor in the media of the three cell lines (expressed as a percentage of the total level measured for that factor, see Methods), with error bars indicating mean ± SD of n = 3 independent

experiments (triplicates each). Significance was determined by one-way ANOVA and post hoc tests (GraphPad Prism 10.3.1). *$p < 0.05$, **$p < 0.01$, ***$p < 0.001$, ****$p < 0.0001$. Cells:

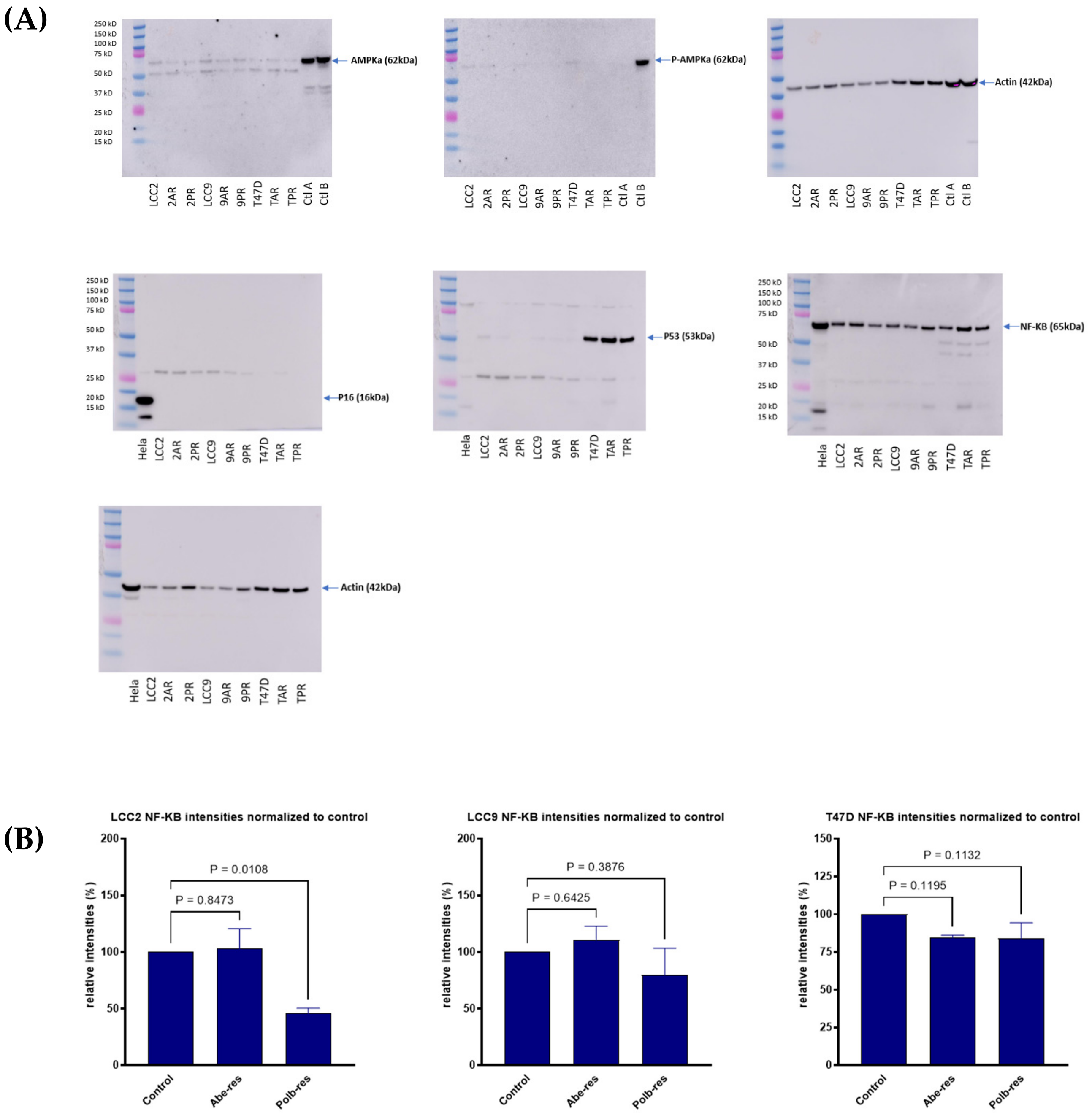

Supplementary Figure S7. Protein Levels of Markers Potentially Involved in SASP. (A) The unprocessed Western blots for protein expression in AR and PR sublines. Protein marker is purchased from BIO-RAD-Precision Plus Protein Dual Color Standards, #1610374, **(B)** Protein quantitation of used as the reference control. AMPKα, p-AMPKα, p16, p53 and NF-κB. Data (mean ± SD) were calculated using ordinary one-way ANOVA (GraphPad Prism 10.3.1) based on three independent biological replicates. β-actin (Sigma, St. Louis, MO) was used as reference control.

(A)

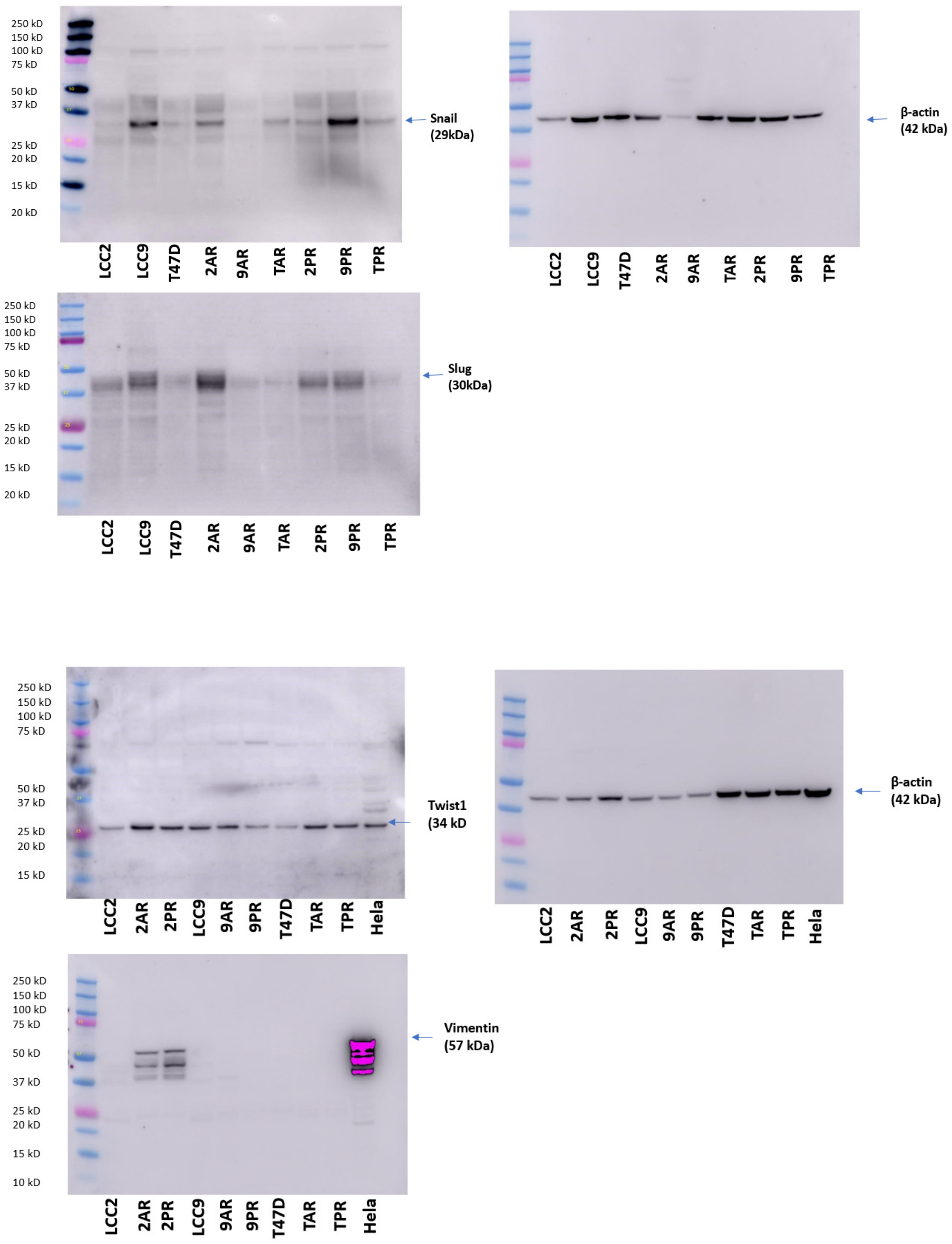
250 kD
150 kD
100 kD
75 kD
50 kD
37 kD
25 kD
20 kD
15 kD
20 kD
Snail
(29kDa)
LCC2
LCC9
T47D
2AR
9AR
TAR
2PR
9PR
TPR
β-actin
(42 kDa)
LCC2
LCC9
T47D
2AR
9AR
TAR
2PR
9PR
TPR
250 kD
150 kD
100 kD
75 kD
50 kD
37 kD
25 kD
20 kD
15 kD
20 kD
Slug
(30kDa)
LCC2
LCC9
T47D
2AR
9AR
TAR
2PR
9PR
TPR
250 kD
150 kD
100 kD
75 kD
50 kD
37 kD
25 kD
20 kD
15 kD
Twist1
(34 kD
LCC2
2AR
2PR
LCC9
9AR
9PR
T47D
TAR
TPR
Hela
β-actin
(42 kDa)
LCC2
2AR
2PR
LCC9
9AR
9PR
T47D
TAR
TPR
Hela
250 kD
150 kD
100 kD
75 kD
50 kD
37 kD
25 kD
20 kD
15 kD
10 kD
Vimentin
(57 kDa)
LCC2
2AR
2PR
LCC9
9AR
9PR
T47D
TAR
TPR
Hela

(B)

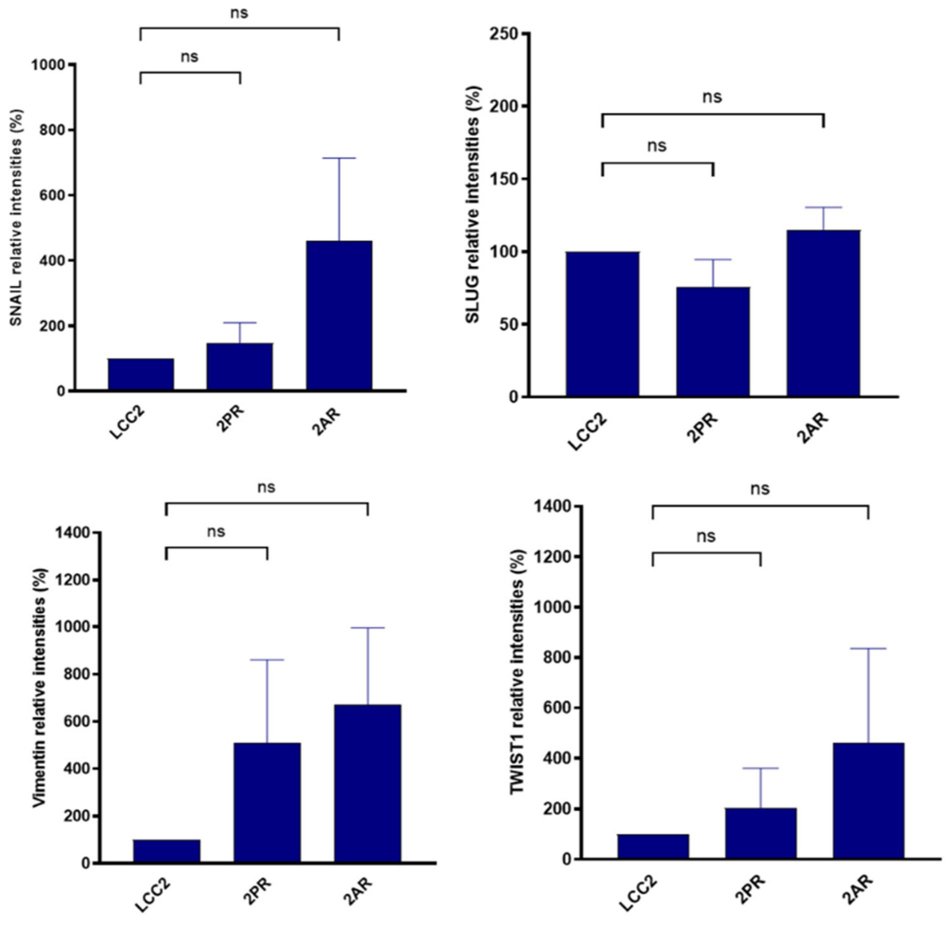

Supplementary Figure S8. Expression of EMT- Transcription Factors in parental and CDK4/6 inhibitor–resistant sublines of ER+ breast cancer. (A) The unprocessed Western blots for protein expression in AR and PR sublines. Protein marker is purchased from BIO-RAD-Precision Plus Protein Dual Color Standards, #1610374. **(B)** Western blot quantification of Snail, Slug, Twist1 and Vimentin in parental (LCC2, LCC9 and T47D), palbociclib-resistant (2PR, 9PR and TPR), and abemaciclib-resistant (2AR, 9AR and TAR) sublines. Bars show protein intensities normalized to β-actin and expressed relative to LCC2 (=100%). Data are mean ± SD from n = 3 independent biological replicates. Statistics: one-way ANOVA with post-hoc testing (GraphPad Prism 10.3.1); ns, not significant.

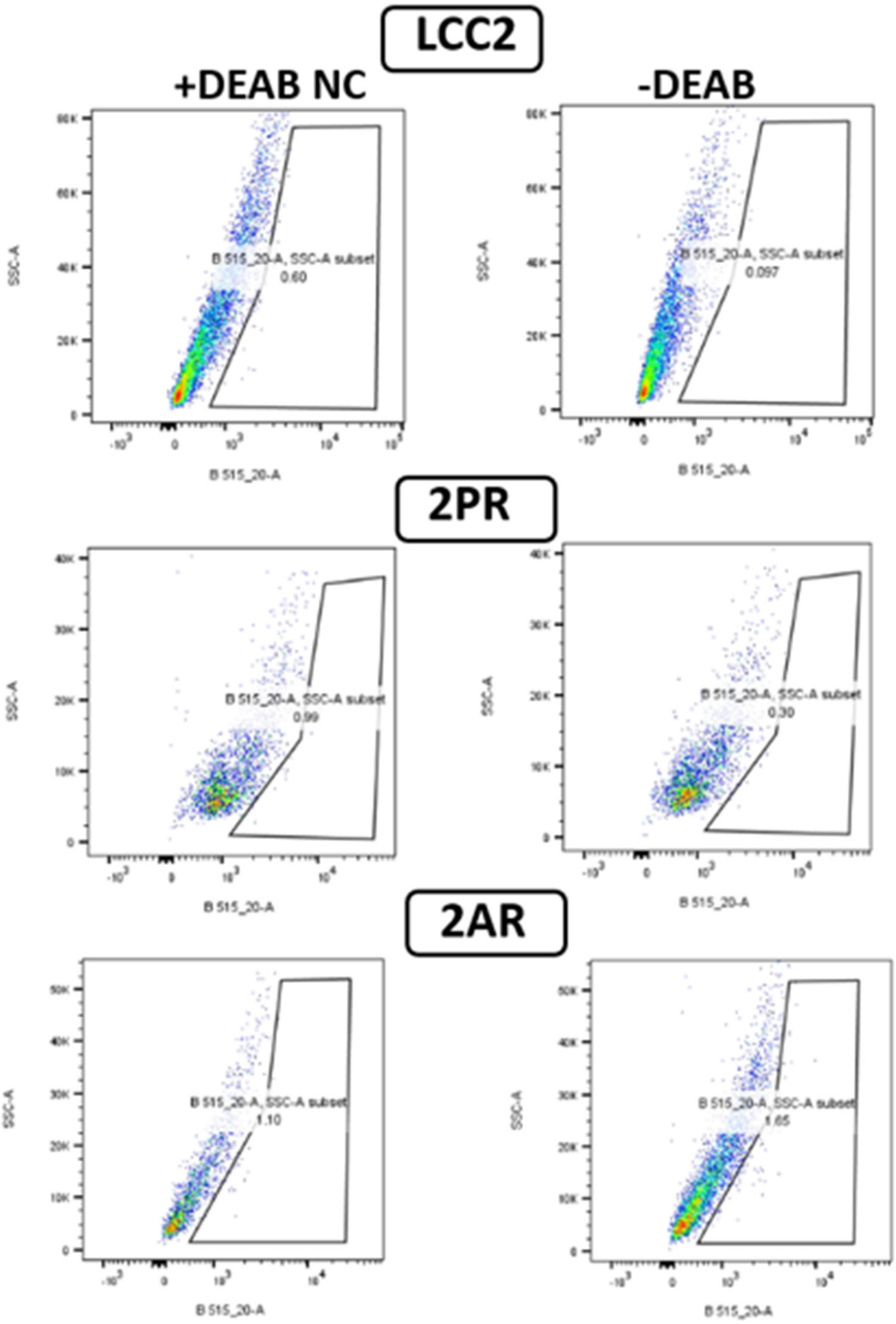

LCC2
+DEAB NC
-DEAB
SSC-A
B 515_20-A
B 515_20-A, SSC-A subset
0.60
0.097
2PR
0.99
0.30
2AR
1.10
1.65

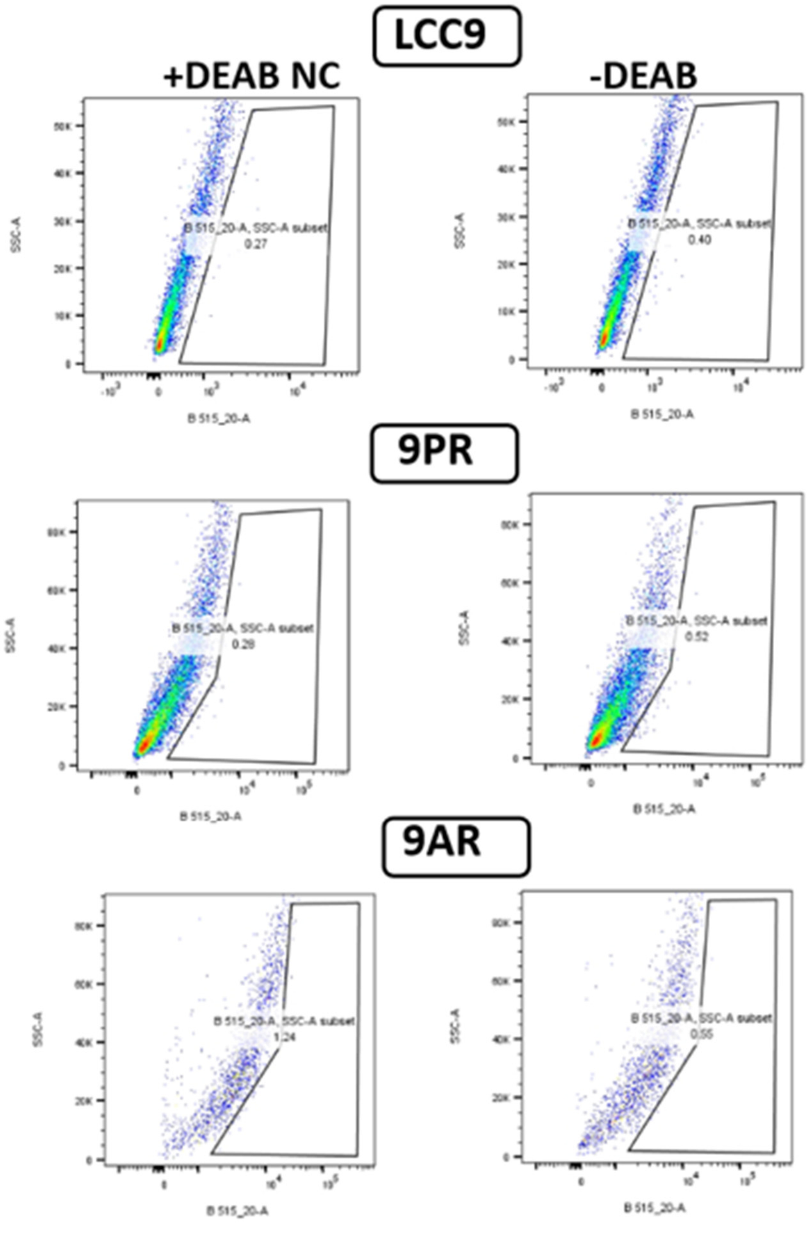
LCC9
+DEAB NC
-DEAB
9PR
9AR
SSC-A
B 515_20-A
B 515_20-A, SSC-A subset
0.27
0.40
0.28
0.52
1.24
0.55

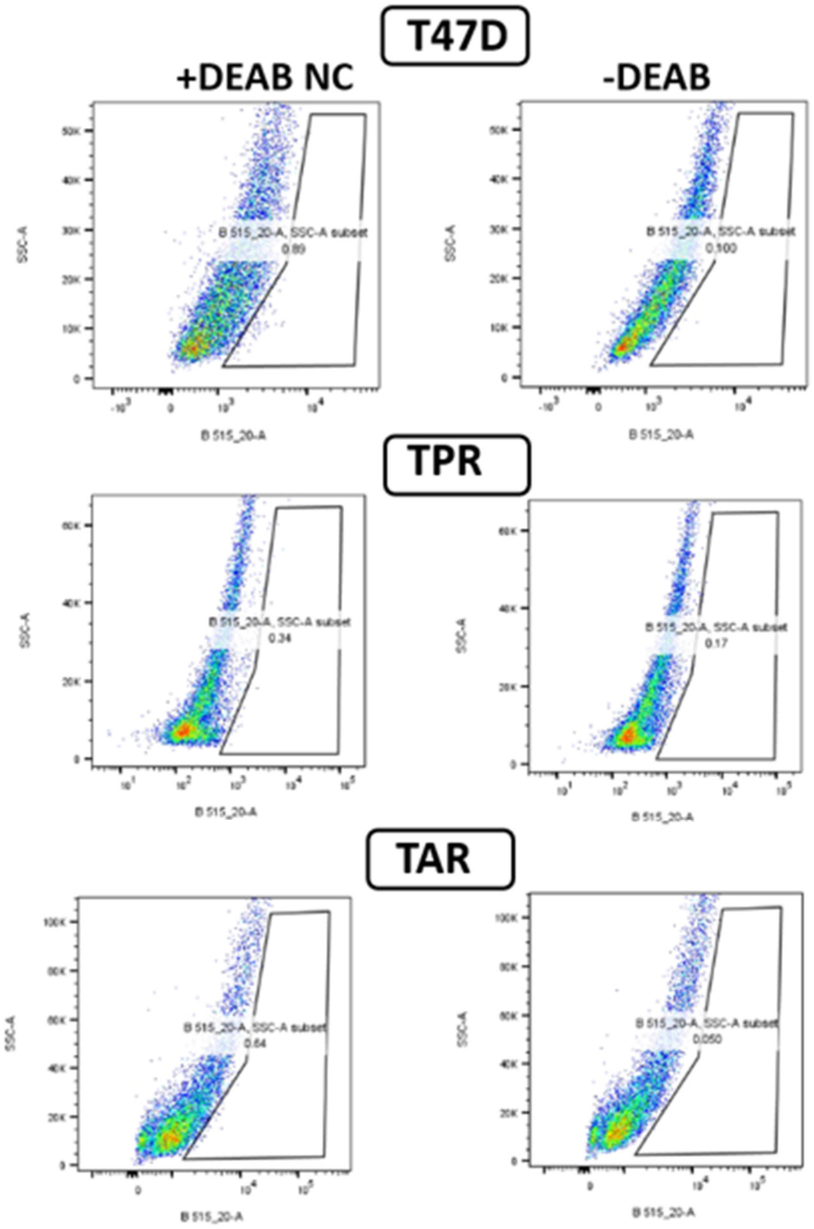

ALDH+ Proportion	LCC2	2PR	2AR	LCC9	9PR	9AR	T47D	TPR	TAR
+DEAB NC	0.60	0.99	1.1	0.4	0.28	1.24	0.89	0.34	0.64
-DEAB	0.097	0.3	1.65	0.27	0.52	0.55	0.1	0.17	0.025

Supplementary Figure S9. ALDH+ populations are not changed in resistant cells compared to their parental counterparts. Representative FACS analysis of palbociclib and abemaciclib-resistint sublines and their parental counterparts using ALDEFLOUR assay. Cells incubated with ALDEFLUOR substrate (BAAA) and the specific inhibitor of ALDH, DEAB, were used to establish the baseline fluorescence of these cells and to define the ALDEFLUOR-positive region. Parental (LCC2, LCC9 and T47D), palbociclib-resistant (2PR, 9PR and TPR), and abemaciclib-resistant (2AR, 9AR and TAR).

Flow-cytometry settings and gating strategy: ALDH activity in parental (LCC2, LCC9 and T47D) and CDK4/6 inhibitor–resistant (2PR, 9PR, TPR, 2AR, 9AR, and TAR) cells was measured using the ALDEFLUOR assay according to the manufacturer's instructions. Samples were acquired with a 488-nm blue laser and the B515/20 detector (FITC-equivalent, B515_20-A parameter) and plotted against SSC-A. Debris and doublets were excluded by FSC/SSC gating, and a live single-cell gate was drawn and used to generate the B515_20-A vs SSC-A dot plots shown for each cell line. In ALDEFLUOR assay, cells treated with the ALDH inhibitor DEAB serve as the negative control to define background fluorescence, while cells without DEAB retain ALDH activity, allowing identification of $ALDH^+$ cells. The $ALDH^+$ region per cell line was defined using the DEAB-treated negative control (+DEAB NC), and the same gate was then applied to the corresponding −DEAB sample to calculate the $ALDH^+$ proportions summarized in the table. Within the live single-cell gate, the numbers of acquired events were: LCC2 +DEAB, 7,045 and LCC2 −DEAB, 6,188; 2PR +DEAB, 2,715 and 2PR −DEAB, 3,006; 2AR +DEAB, 2,007 and 2AR −DEAB, 5,029; 9AR +DEAB, 2,022 and 9AR −DEAB, 2,007. For all LCC9, 9PR, T47D, TPR and TAR samples, 10,000 single-cell events were acquired per condition (+DEAB and −DEAB). This figure displays the same event numbers underneath each dot's plot.

Notes common to Supplementary Figures

Cell lines: Palbociclib and abemaciclib-resistant sublines of LCC2, LCC9 and T47D were generated as described in Materials and Methods.

Normalization: ELISA data were normalized to "% of total per analyte" within plate to allow cross-analyte comparison; TMRE was normalized to parental line (=100%).

Statistics: Normality was tested where appropriate; unless otherwise stated, one-way ANOVA (Tukey) was used using GraphPad Prism 10.3.1 software. Exact n and p values are indicated in the figure panels or in the main text.

Reagents/instrumentation: ELISAs (Thermo Fisher ready-to-use plates, R&D Systems, Biotechne or Chondrex, Inc.); Seahorse XF Analyzer (Agilent) with standard injection sequences; TMRE assay (Abcam) according to manufacturer's protocol as described in Materials and Methods.

Abbreviations: AR, abemaciclib-resistant; PR, palbociclib-resistant; ECAR, extracellular acidification rate; OCR, oxygen consumption rate; $\Delta\Psi m$, mitochondrial membrane potential; SASP, senescence-associated secretory phenotype.
